# Efficient Selective Sorting of Semiconducting Carbon Nanotubes Using Ultra-Narrow-Band-Gap Polymers

**DOI:** 10.1021/acsami.2c07158

**Published:** 2022-08-09

**Authors:** Wytse Talsma, Gang Ye, Yuru Liu, Herman Duim, Sietske Dijkstra, Karolina Tran, Junle Qu, Jun Song, Ryan C. Chiechi, Maria Antonietta Loi

**Affiliations:** † Zernike Institute for Advanced Materials, University of Groningen, Nijenborgh 4, 9747 AG Groningen, The Netherlands; ‡ Center for Biomedical Optics and Photonics (CBOP) & College of Physics and Optoelectronic Engineering, Key Laboratory of Optoelectronic Devices and Systems, Shenzhen University, Shenzhen 518060, PR China; § Stratingh Institute for Chemistry, University of Groningen, Nijenborgh 4, 9747 AG Groningen, The Netherlands; ∥ Department of Chemistry and Carbon Electronics Cluster, North Carolina State University, Raleigh, North Carolina 27695-8204, United States

**Keywords:** low-band-gap conjugated polymers, polar side chain, SWCNT FET, sorting SWCNTs, polymer wrapping

## Abstract

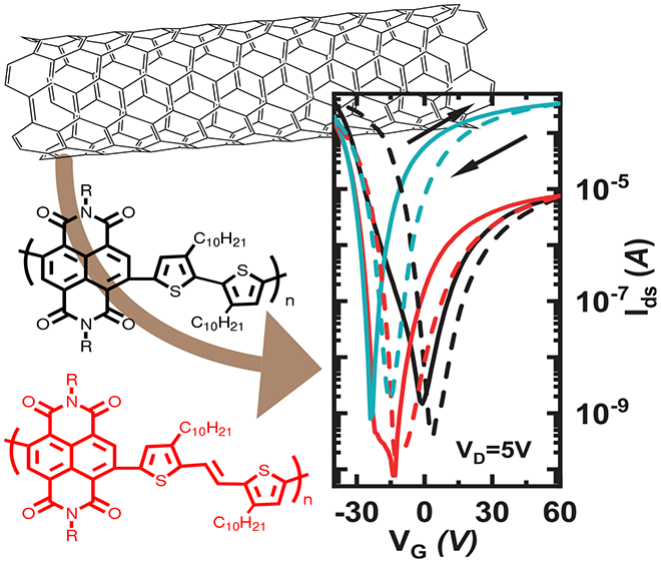

Conjugated polymers with narrow band gaps are particularly useful for sorting and discriminating semiconducting single-walled carbon nanotubes (s-SWCNT) due to the low charge carrier injection barrier for transport. In this paper, we report two newly synthesized narrow-band-gap conjugated polymers (**PNDITEG-TVT** and **PNDIC8TEG-TVT**) based on naphthalene diimide (NDI) and thienylennevinylene (TVT) building blocks, decorated with different polar side chains that can be used for dispersing and discriminating s-SWCNT. Compared with the mid-band-gap conjugated polymer **PNDITEG-AH**, which is composed of naphthalene diimide (NDI) and head-to-head bithiophene building blocks, the addition of a vinylene linker eliminates the steric congestion present in head-to-head bithiophene, which promotes backbone planarity, extending the π-conjugation length and narrowing the band gap. Cyclic voltammetry (CV) and density functional theory (DFT) calculations suggest that inserting a vinylene group in a head-to-head bithiophene efficiently lifts the highest occupied molecular orbital (HOMO) level (−5.60 eV for **PNDITEG-AH**, −5.02 eV for **PNDITEG-TVT**, and −5.09 eV for **PNDIC8TEG-TVT**). All three polymers are able to select for s-SWCNT, as evidenced by the sharp transitions in the absorption spectra. Field-effect transistors (FETs) fabricated with the polymer:SWCNT inks display p-dominant properties, with higher hole mobilities when using the NDI-TVT polymers as compared with **PNDITEG-AH** (0.6 cm^2^ V^–1^ s^–1^ for HiPCO:**PNDITEG-AH**, 1.5 cm^2^ V^–1^ s^–1^ for HiPCO:**PNDITEG-TVT**, and 2.3 cm^2^ V^–1^ s^–1^ for HiPCO:**PNDIC8TEG-TVT**). This improvement is due to the better alignment of the HOMO level of **PNDITEG-TVT** and **PNDIC8TEG-TVT** with that of the dominant SWCNT specie.

## Introduction

Single-walled carbon nanotubes (SWCNT) are one of the most promising next-generation electronic materials for field-effect transistors (FETs),^1−5^ logic circuits,^6−10^ sensors,^11,12^ flexible electronics,^13−15^ microprocessors,^16,17^ and other applications.^18−21^ Among these applications, FETs based on semiconducting SWCNT (s-SWCNT) are attractive because of their high charge carrier mobility, low dimensionality, and the possibility for high-density integration and low-cost solution processability.^22−24^ Indeed, pure s-SWCNT FETs have demonstrated performance superior to that of silicon.^25^ However, as-synthesized SWCNT are a mixture of roughly 2:1 s-SWCNT and m-SWCNT (metallic SWCNT), which limits their application in devices.^26,27^

**Figure 1:**
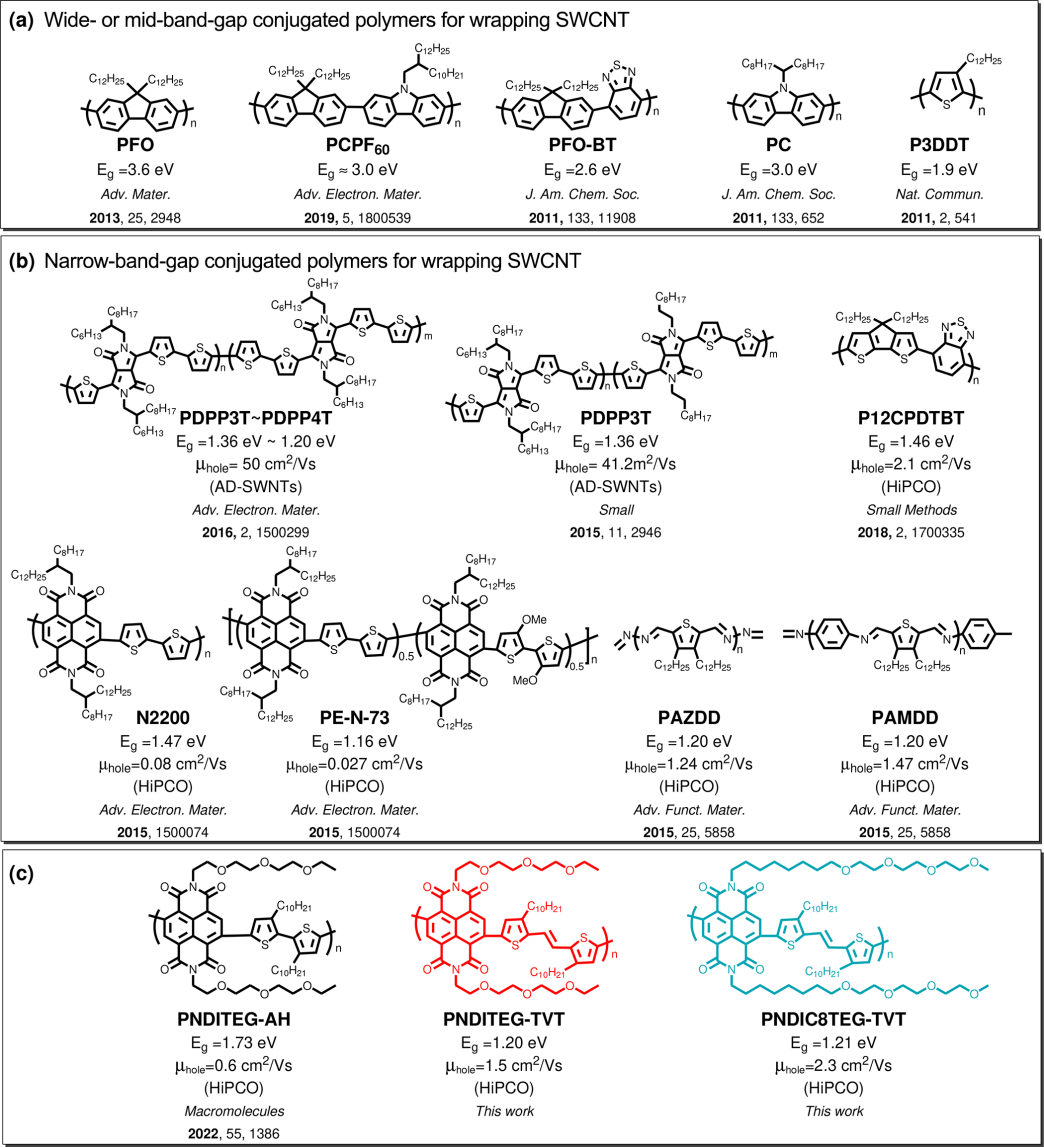
Chemical structures of (a) representative wide- or mid-band-gap conjugated polymers for selecting and sorting SWCNTs reported in the literature and (b) narrow-band-gap conjugated polymers for selecting and sorting SWCNTs reported so far. The mentioned mobilities are for s-SWCNT FETs (c) naphthalene diimide based low-band-gap conjugated polymers presented in this work.^31,33,34,36−41,53^

To overcome these issues, several approaches to isolate pure s/m-SWCNT have been developed. Among these are (i) density gradient ultracentrifugation (DGU),^28^ (ii) size-exclusion chromatography methods,^29^ and (iii) noncovalent selective sorting of s-SWCNT by conjugated polymers.^22−24^ The latter technique has received significant attention because of its high dispersion efficiency, selectivity, and low cost.^27^ Since the pioneering work of Nish et al. in 2007, where polyfluorene was used to select semiconducting carbon nanotubes at a low concentration,^30^ many conjugated polymers able to disperse and sort s-SWCNT selectively have been reported.^22,27^ Most of those conjugated polymers are fluorene-,^30,31^ carbazole-,^32,33^ and thiophene-based polymers,^34,35^ with a highest occupied molecular orbital (HOMO) and lowest unoccupied molecular orbital (LUMO) separation (band gap) of about 2 or 3 eV (see Figure 1a).^23,24,27^ From the perspective of device fabrication, the advantage of using conjugated polymers to wrap s-SWCNT selectively is that the noncovalently bound polymers do not necessarily affect the intrinsic charge transport of the SWCNT. However, in practice, polymers with relatively large band gaps create energetic barriers to intertube transport in the solid state, deleteriously affecting the electrical performance of devices. This barrier is the result of an energy mismatch between the s-SWCNT and the polymer; thus, to reduce the energetic barrier between the polymer and nanotubes, the band energies (HOMO/LUMO levels) of the wrapping polymer need to match those of the s-SWCNT. While there are some instances in which it might be desirable to promote hole or electron transport, narrow-band-gap polymers match both levels, facilitating both electron and hole transport, making the s-SWCNT networks more generalizable to device applications.

There are few examples of narrow-band-gap conjugated polymers that demonstrably disperse SWCNT selectively (see Figure 1b).^36−40^ In our previous work, we showed that the naphthalene-diimide (NDI) based, donor–acceptor (D-A), narrow-band-gap conjugated polymers N2200 (1.46 eV) and PE-N-73 (1.16 eV) dispersed HiPCO SWCNTs. However, due to the weak interaction with SWCNTs, the yield was limited, resulting in inks of low concentration.^39^ Because of the low concentration of the tested solution, FETs made from these inks did not perform as well as other polymers (e.g. P3DDT and PFO). Encouragingly, the HOMO level of PE-N-73 is better aligned with that of s-SWCNTs, resulting in reduced IV hysteresis.^1,39^ That work motivated us to design new narrow-band-gap conjugated polymers that maximize interactions with SWCNTs to disperse and select s-SWCNTs.

Very recently, we addressed the weak interaction between D–A conjugated polymers and SWCNTs by introducing polar triethylene glycol side chains on the D–A conjugated polymer backbone. We demonstrated the wrapping ability of the mid-band-gap, D-A conjugated polymer (**PNDITEG-AH**), comprising NDI and head-to-head bithiophene, which is significantly improved by including polar triethylene glycol side chains as compared to alkyl side chains.^41^ For this work, we partly make use of the same data set regarding this specific polymer (**PNDITEG-AH**) and thereon-based devices.

Inspired by these results, we developed new NDI-based, D-A, narrow-band-gap conjugated polymers, **PNDITEG-TVT** and **PNDIC8TEG-TVT**, which include a vinylene linker between the head-to-head bithiophene moiety of the **PNDITEG-AH** backbone to increase the effective conjugation length by planarizing the backbone, with the goal of promoting intertube transport in the resulting inks. It has been reported that backbone planarity and the aromatic surface area of the polymer promote better π–π interactions with SWCNT, thus enhancing the s-SWCNT wrapping capability.^37,38^ Importantly, **PNDITEG-TVT** and **PNDIC8TEG-TVT** remain flexible due to the steric hindrance of the NDI with the adjacent thiophene, thus avoiding the formation of a rigid conjugated polymer backbone that would disfavor the wrapping process energetically, reducing the yield for the selection of s-SWCNTs.^40^

In this paper, we describe the design and synthesis of two narrow-band-gap conjugated polymers by copolymerization of NDI moieties with thienylennevinylene (TVT) decorated with polar side chain for dispersing and discriminating s-SWCNTs. Both polymers demonstrate the capacity of selectively dispersing s-SWCNTs in good yields. Absorption measurements of the resulting inks reveal that **PNDITEG-AH** exhibits the highest yield for s-SWCNTs dispersion due to higher flexibility of the backbone, while the planar structure of **PNDITEG-TVT** leads to a modest decrease of 12% in dispersion ability. FETs fabricated from either of these polymer-wrapped s-SWCNT inks exhibit excellent charge-transport properties due to narrow band gap lowering the energetic barrier for intertube charge-transport. This work clearly demonstrates the critical role of backbone flexibility and planarity, affording a deeper understanding of the relationship between polymer structure and the wrapping and selecting of s-SWCNTs.

## Results and Discussion

### Synthesis and Characterization

**Figure 2:**
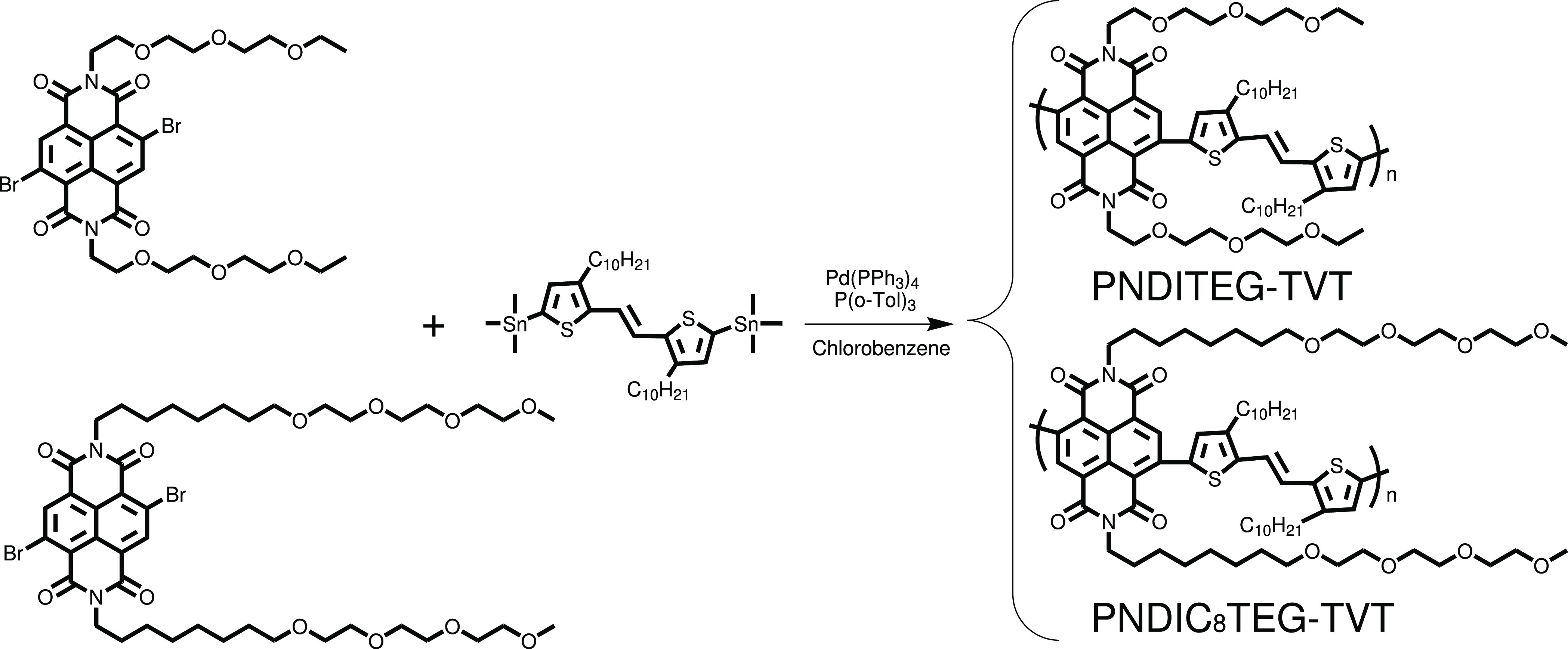
Synthesis routes to naphthalene diimide and thienylennevinylene based low-band-gap conjugated polymers **PNDITEG-TVT** and **PNDIC8TEG-TVT**, used in this work.

The synthesis routes and chemical structures of the two new conjugated polymers that were used to study the dispersion and discrimination of semiconducting carbon nanotubes are shown in Figure 2. The synthesis of **PNDITEG-AH** was carried out according to literature procedures.^41^ The monomers were synthesized and purified according to published procedures with slight modifications (see the Supporting Information for details).^42−44^ Both polymers were synthesized by a typical palladium-catalyzed Stille polycondensation of symmetrical dibromo naphthalene diimide and distannyl thienylennevinylene monomers. Polymers were obtained by refluxing the degassed polymerization mixture for 3 days. After polymerization, the crude polymers were collected by precipitation in methanol. Impurities and low-molecular-weight fractions were removed by continuous extraction with methanol followed by hexane and then chloroform in a Soxhlet extractor. Finally, the polymer solution in chloroform was concentrated, redissolved, precipitated into cold methanol, collected, and dried in vacuum. The yields for **PNDITEG-AH**, **PNDITEG-TVT**, and **PNDIC8TEG-TVT** are 74, 87, and 90%, respectively. The target conjugated polymer structures were characterized by ^1^H NMR, FT-IR, MALDI-TOF-MS, and GPC (Figures S16–S21 and S23–S25 and Table S1). The thermal properties of these three polymers were evaluated by thermogravimetric analysis (TGA) and differential scanning calorimetry (DSC). The temperature of 5% weight-loss was selected as the onset point of decomposition (*T*_d_). As shown in Figure S26 a, all the polymers show excellent stability with a decomposition temperature of 361 °C for **PNDITEG-AH**, 373 °C for **PNDITEG-TVT**, and 407 °C for **PNDIC8TEG-TVT**. The DSC curves of **PNDITEG-TVT** and **PNDIC8TEG-TVT** reveal no distinct phase transition from room temperature to 300 °C, while **PNDITEG-AH** shows a melting transition at 239 °C in the second heating cycle (Figure S26 b).

### Optical and Electrochemical Properties

**Figure 3:**
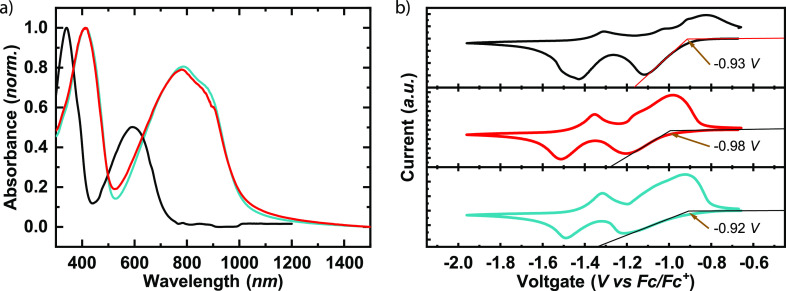
(a) Normalized absorption spectra of pristine **PNDITEG-AH** (black), **PNDITEG-TVT** (red), and **PNDIC8TEG-TVT** (turquoise) as drop-cast (from CHCl_3_) films. (b) Cyclic voltammograms of thin films of **PNDITEG-AH** (black), **PNDITEG-TVT** (red), and **PNDIC8TEG-TVT** (turquoise) versus Fc/Fc^+^ on a glassy carbon working electrode immersed in 0.1 mol L^–1^*n*-Bu_4_NPF_6_ acetonitrile solution at 100 m V s^–1^.

Figure 3a shows the absorption spectra of thin films of **PNDITEG-AH**, **PNDITEG-TVT**, and **PNDIC8TEG-TVT**. All these polymers show two distinct absorption bands that can be assigned to the high energy π–π* transition (300–400 nm for **PNDITEG-AH**, 300–500 nm for **PNDITEG-TVT** and **PNDIC8TEG-TVT**) and the broad, low-energy intramolecular charge-transfer transition (400–700 nm for **PNDITEG-AH**, 550–1000 nm for **PNDITEG-TVT** and **PNDIC8TEG-TVT**) originating from its donor–acceptor structure.^42^**PNDITEG-TVT** and **PNDIC8TEG-TVT** have almost identical spectra due to their shared backbone structure, with only subtle changes in their side chains. Their spectra are red-shifted about 193 nm with respect to that of **PNDITEG-AH**, indicating that the insertion of vinylene moieties reduces the steric hindrance of the head-to-head bithiophene units, promoting backbone planarity and extending the π-conjugation length.^45−50^ The absorption onsets (λ_onset_^film^) in films of **PNDITEG-AH**, **PNDITEG-TVT**, and **PNDIC8TEG-TVT** are 718, 1035, and 1020 nm, and their corresponding optical band gap are calculated to be 1.73, 1.20, and 1.21 eV, respectively (see Table 1).

**Table 1:** Summary of the Photophysical Properties, Electrochemical Properties, Energy Levels, and Molecular Weights of **PNDITEG-AH**, **PNDITEG-TVT**, and **PNDIC8TEG-TVT**

physical property	**PNDITEG-AH**	**PNDITEG-TVT**	**PNDIC8TEG-TVT**
λ_max_^sol^ (nm)	335, 560	390, 690	390, 690
λ_max_^film^ (nm)	340, 593	411, 783	411, 785
λ_onset_^film^ (nm)	718	1035	1020
*E*_g_^opt.^^a^ (nm)	1.73	1.20	1.21
*E*_onset_^red^ (V)	–0.93	–0.98	–0.92
LUMO (eV)^b^	–3.87	–3.82	–3.88
HOMO (eV)^c^	–5.60	–5.02	–5.09
LUMO (eV)^d^	–3.64	–3.56	–3.48
HOMO (eV)^d^	–6.15	–5.45	–5.41
*M*_n_ (g/mol)^e^	9014	14246	15662
*M*_w_ (g/mol)^e^	14568	54452	55990
PDI^e^	1.51	3.83	3.57
DP (degrees of polymerization)^e^	9	13	12

a *E*_g_^opt.^ = 1240/λ_onset_^film^.

b Calculated from CV: *E*_LUMO_ = -(4.80 + *E*_onset_^red^) eV.

c Calculated from *E*_LUMO_ and *E*_g_^opt.^: *E*_HOMO_ = *E*_LUMO_ + *E*_g_^opt^.

d From DFT calculations.

e From GPC analysis.

We determined the energy levels of **PNDITEG-AH**, **PNDITEG-TVT** and **PNDIC8TEG-TVT** by cyclic voltammetry (CV); details can be found in the Supporting Information. The results are shown in Figure 3b and summarized in Table 1. All polymers exhibit two quasi-reversible reduction waves, corresponding to n-doping (reduction) as a result of the electron-deficient nature of the NDI moieties. The onset reduction potentials (*E*_onset_^Red.^) of **PNDITEG-AH**, **PNDITEG-TVT**, and **PNDIC8TEG-TVT** are −0.93, −0.98, and −0.92 V, respectively. The energy levels of the polymers were calculated using the equation *E*_LUMO_ = −(4.80 + *E*_onset_^Red.^) eV, where 4.80 represents the HOMO energy level of ferrocene against vacuum. Accordingly, the LUMO levels of polymers are −3.87, −3.82, and −3.88 eV, respectively. On the basis of the optical band gap and LUMO levels, the calculated HOMO levels of **PNDITEG-AH**, **PNDITEG-TVT**, and **PNDIC8TEG-TVT** are −5.60, −5.02, and −5.09 eV, respectively. These results reveal that in addition to planarizing the backbone the incorporation of vinylene moieties into the polymer backbone pushes the HOMO level up, which should further facilitate hole transport between the polymer and s-SWCNT.

Density functional theory (DFT) calculations were carried out on model compounds for **PNDITEG-AH**, **PNDITEG-TVT** and **PNDIC8TEG-TVT** to elucidate further the effect of the vinylene linker on the backbone planarity and energy levels of the frontier orbitals at the B3LYP/6-311G(d) level using Gaussian 16.^51^ In Figure S29, the molecular orbital distributions for model compounds of **PNDITEG-AH**, **PNDITEG-TVT**, and **PNDIC8TEG-TVT** are shown. All the LUMOs are localized on the central NDI unit. In contrast, the HOMOs are located on the TVT moiety in **PNDITEG-TVT** and **PNDIC8TEG-TVT** and on bithiophene in **PNDITEG-AH**. Figure S30 shows that the calculated energy gaps of **PNDITEG-TVT** and **PNDIC8TEG-TVT** are lower than that of **PNDITEG-AH**, which is consistent with the optical band gap results. Interestingly, the introduction of vinylene linker into the head-to-head bithiophene significantly increased the HOMO energy, with little change in the LUMO energy, resulting in a decreased band gap. This is in agreement with our CV measurements.

The dihedral angle between thiophene and thiophene in **PNDITEG-AH** is 109.0°, which is considerably larger than that of the thiophene adjacent to the vinylene moieties in **PNDITEG-TVT** (4.8 and 1.7°) and **PNDIC8TEG-TVT** (4.7 and 1.6°). In addition, the dihedral angle between the NDI unit and adjacent thiophene is also reduced, from 61.9° for **PNDITEG-AH** to 39.5° for **PNDITEG-TVT** and to 40.1° for **PNDIC8TEG-TVT** (see Figures S31–S33 and Table S2).

### s-SWCNT Ink Characterization

**Figure 4:**
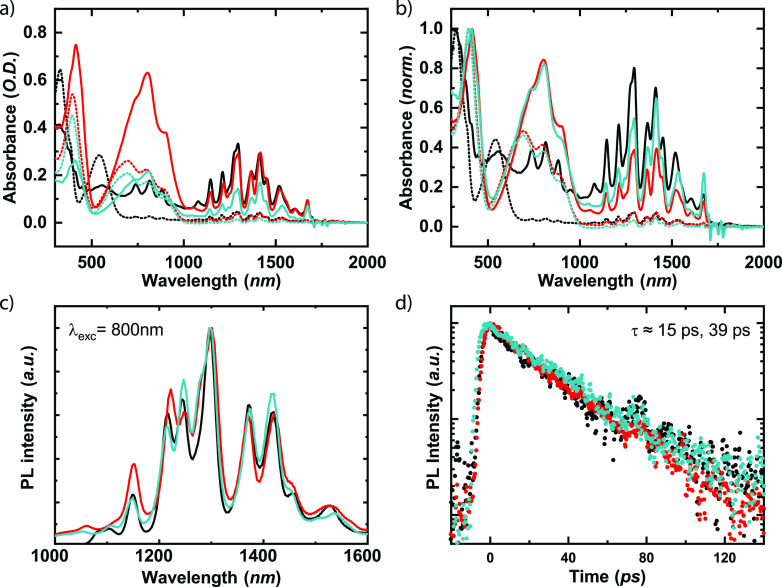
(a) Absorption spectra (in O.D.) of HiPCO:**PNDITEG-AH** (black), HiPCO:**PNDITEG-TVT** (red), and HiPCO:**PNDIC8TEG-TVT** (turquoise) inks as obtained directly from the first discrimination (dotted lines) and after the enrichment procedure (solid lines). (b) Absorption spectra normalized to the free polymer peak. (c) Normalized steady-state photoluminescence of the HiPCO:**PNDITEG-AH** (black), HiPCO:**PNDITEG-TVT** (red), and HiPCO:**PNDIC8TEG-TVT** (turquoise). (d) Photoluminescence decay of the 1140 nm PL peak of the investigated samples. No discernible differences in lifetimes were found between the SWCNT solutions obtained with the different polymers; a fit of the measurements with *I* = ∑_*i*=1_^2^*A*_*i*_ exp((−*t*)/(τ_*i*_)) yields lifetimes τ_1_ = 15 ps (43%) and τ_2_ = 39 ps (57%).

Having established that inserting a vinylene linker can improve planarity and raise the HOMO level of narrow-band-gap polymers, we tested the performance of these polymers by using them to disperse and select s-SWCNTs. Absorption spectroscopy is the most common and facile method to evaluate the quality of s-SWCNT/polymer inks. Figure 4a shows the absolute absorbance spectra of the polymer wrapped HiPCO-SWCNT inks after the first sonication and ultracentrifugation enrichment (see the Experimental Section). Dashed lines correspond to the spectra as obtained directly after first dispersal of the s-SWCNT. The solid lines are the spectra obtained for the enriched s-SWCNT inks, after two ultracentrifugation runs.^52^ As discussed previously, the absorption peaks of **PNDITEG-TVT** and **PNDIC8TEG-TVT** are located from 500 to 1000 nm, which upon aggregation of the polymer chains exhibits distinct, additional features (see Figure S28). The narrow absorption peaks in the near-infrared region (1100–1700 nm) are evidence of the successful sorting of s-SWCNTs, corresponding to the first (*S*_11_) electronic transitions of the different species present in the sample. These data confirm that **PNDITEG-AH**, **PNDITEG-TVT**, and **PNDIC8TEG-TVT** are able to discriminate and disperse s-SWCNTs selectively.

As expected, the *S*_11_ peaks are significantly enhanced after the enrichment procedure. From these intensities, it is clear that inks prepared using **PNDIC8TEG-TVT** yield the lowest density of s-SWCNT compared with **PNDITEG-TVT** and **PNDITEG-AH** in both discrimination-only and enriched inks. By contrast, there is little difference between the optical density of the s-SWCNTs obtained with **PNDITEG-TVT** and **PNDITEG-AH**, demonstrating the predicted effects of the vinylene moieties described above. We note that to be able to compare the absolute yields of s-SWCNT selected by each polymer directly we did not dilute the dispersion inks to an equal optical density.

To highlight the relative differences in yield with respect to the amount of polymer chains, we normalized the absorbance spectra to the free polymer peak (at 327 nm for **PNDITEG-AH** and 392–410 nm for **PNDITEG-TVT** and **PNDIC8TEG-TVT**), as shown in Figure 4b. Relative to this standard, **PNDITEG-AH** yields the highest amount of s-SWCNT after both the selection and enrichment steps. Comparing **PNDIC8TEG-TVT** and **PNDITEG-TVT**, the first produces a smaller amount of s-SWCNTs for first sorted inks, as can be seen in the *S*_11_ (1100–1700 nm) region. However, interestingly, after the crucial enrichment step, a different trend emerges: The **PNDIC8TEG-TVT** sample yields a higher density of s-SWCNT, and the **PNDITEG-TVT** sample contains a lower amount of s-SWCNTs relative to free polymer. This indicates that it is more difficult to remove excess **PNDITEG-TVT** from the inks, resulting in a lower overall wrapping efficiency. The change in the ratio between the two polymer absorption peaks is significant, as the lower energy peak is indicative of aggregation on the SWCNT walls, while the high energy peak is a finger print of the free polymer chains.^40^ In particular, after the second centrifugation, the low energy peak becomes more prominent, indicating that the polymer indeed is coiling around the s-SWCNT.

The absorption data demonstrate that the more planar polymers **PNDITEG-TVT** and **PNDIC8TEG-TVT** have a lower capability of dispersing (but not selecting for) s-SWCNT than the less planar **PNDITEG-AH**. The higher ability of **PNDITEG-AH** for dispersing s-SWCNT is likely a result of the twist in the head-to-head bithiophene moiety that favors a helical conformation for wrapping nanotubes.^40,53−55^ The minor difference between **PNDITEG-AH** and **PNDITEG-TVT** indicates that inserting a vinylene linker into **PNDITEG-AH** (thus forming **PNDITEG-TVT**) did not significantly sacrifice wrapping capability. Although the planar polymer **PNDITEG-TVT** is less prone to form a helical conformation to wrap s-SWCNT as compared with **PNDITEG-AH**, the extended π-conjugation gives rise to a strong π–π intermolecular interaction, which enhances π–π interaction between the polymer chains and the s-SWCNT.^24,37,38^ Therefore, **PNDITEG-TVT** remains capable of wrapping the s-SWCNT, but the lower band gap compared to **PNDITEG-AH** should be beneficial to charge transport.

It has been demonstrated that the side chains are also very important for the wrapping of SWCNTs.^53^ Therefore, since **PNDITEG-TVT** and **PNDIC8TEG-TVT** consist of the same π-conjugated backbone, the difference in dispersion yield should be a direct result from the variation in the side chain. Inserting an alkyl chain segment between the glycol side chain and backbone not only increases the solubility of the polymer but also reduces the polarity of **PNDIC8TEG-TVT**, which in turn results in a lower interaction with s-SWCNTs.^23,24,27^ Thus, **PNDIC8TEG-TVT** exhibits the lowest wrapping ability during selection. However, during the enrichment procedure, the excess (free) polymer in the supernatant is more easily removed in the case of **PNDIC8TEG-TVT** as compared with **PNDITEG-TVT**, which is likely due to the more flexible side chain of **PNDIC8TEG-TVT**, which increases its solubility. Since the polymers have a similar molecular weight and the amount used for preparing the solutions is kept constant, the total number of polymer chains within each solution is comparable.

In Figure 4c, the normalized steady-state photoluminescence (PL) spectra of the inks are shown. Using **PNDITEG-AH** as a reference, we can see that **PNDITEG-TVT** shows a slight preference for emission from high band gap SWCNTs. This is in contrast with **PNDIC8TEG-TVT**, which shows slightly more intense emission at lower energies.

The small difference in intensity of the PL, considering that the absorption spectra of the three dispersions are almost identical, indicates subtle variation in the degree of energy transfer among SWCNTs of different chirality. This difference is further confirmed by time-resolved PL in Figure 4d, where the 1140 nm transition shows similar decay for all inks, with extracted lifetimes of τ_1_ = 15 ps (43%) and τ_2_ = 39 ps (57%). Since the lifetime is typically strongly quenched in the presence of metallic species and proximity with SWCNT of different chirality, the data indicate that the inks fabricated from the new polymers result in a similar degree of s-SWCNT individualization.^56^ It is important to note that this is not always the case; we have previously demonstrated that in case of SWCNT wrapped with polythiophenes, the formation of twins is favored with strong consequences for the PL lifetime.^57^

### Thin-Film Transistor Performance

**Figure 5:**
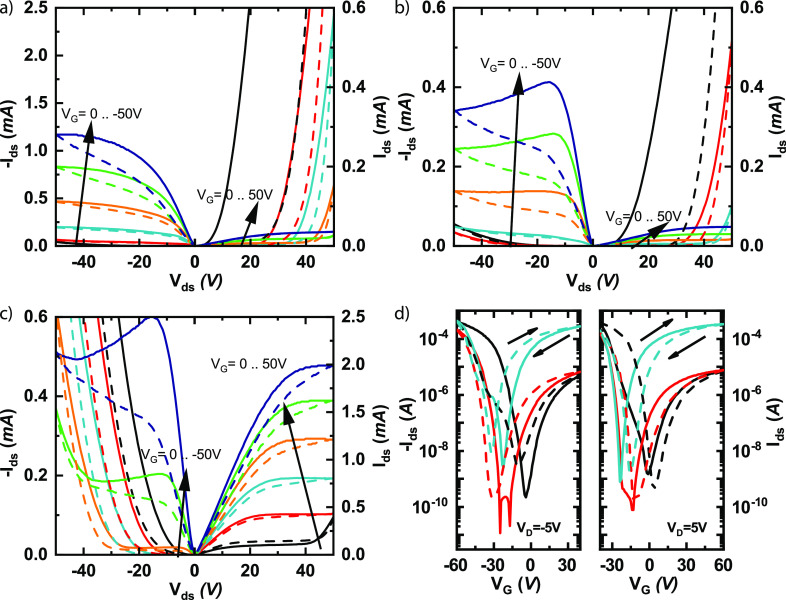
Comparison of electrical characteristics for the polymer wrapped s-SWCNT inks. (a–c) FET output characteristics for the examined inks for (a) a device fabricated from HiPCO:**PNDITEG-AH** (Adapted from our previous work^41^), (b) HiPCO:**PNDITEG-TVT**, and (c) HiPCO:**PNDIC8TEG-TVT**. The arrows represent an increase in gate bias from 0 to ±50 V in steps of 10 V. (d) FET transfer characteristics for HiPCO:**PNDITEG-AH** (black), HiPCO:**PNDITEG-TVT** (red), and HiPCO:**PNDIC8TEG-TVT** (turquoise). The arrows represent the sweep directions.

To evaluate the quality of the s-SWCNT:polymer inks and probe their charge transport properties, we fabricated bottom-gate and bottom-contact field-effect transistors (FETs). The enriched polymer sorted s-SWCNT inks were blade-cast onto the substrate with lithographically defined electrodes. Figure 5 displays the electrical characteristics of a representative set of FET devices made from the enriched polymer wrapped HiPCO s-SWCNT inks. There is a peculiar shift from p-type dominated toward more ambipolar, n-type dominated transport, going from HiPCO:**PNDITEG-AH** to HiPCO:**PNDITEG-TVT** to HiPCO:**PNDIC8TEG-TVT**. As shown in Figure 5a, the **PNDITEG-AH**-sorted s-SWCNT transistors exhibited p-dominated output characteristics. While in Figure 5b, HiPCO:**PNDITEG-TVT**-based FET still displayed p-dominated output characteristics, and slightly more electrons are present in the channel. In Figure 5c, the HiPCO:**PNDIC8TEG-TVT**-based FET displayed n-dominated output characteristics. The electron current for the HiPCO:**PNDITEG-TVT** device is almost 1 order of magnitude smaller as compared with HiPCO:**PNDITEG-AH**. Therefore, indicating that the electron-trapping mechanisms play relatively a more significant role in the transport properties, resulting in a larger relative hysteresis and eventually saturation (at about −15 V) of the channel current. The increased relative trapping together with charge carrier recombination can also explain the decrease of the current after saturation.

The p-type charge carrier dominated HiPCO:**PNDITEG-AH** ink exhibit the highest hole saturation current, while the HiPCO:**PNDITEG-TVT** and HiPCO:**PNDIC8TEG-TVT** inks exhibit similar hole current saturation. However, the electron current for the devices made from the enriched **PNDIC8TEG-TVT**-sorted s-SWCNT inks is 40 times larger in magnitude as compared with enriched **PNDITEG-TVT**-sorted s-SWCNT inks. Figure 5d shows the *I*_D_–*V*_G_ transfer characteristics of all three devices. The FETs obtained from **PNDITEG-AH**-sorted s-SWCNT exhibit a hole mobility of 0.82 cm^2^ V^–1^ s^–1^ (on–off ratio ≈ 9 × 10^6^) and an electron mobility of 0.02 cm^2^ V^–1^ s^–1^ (on–off ratio ≈ 8 × 10^3^). The FETs obtained from **PNDITEG-TVT**-sorted s-SWCNT exhibit a hole mobility of 1.52 cm^2^ V^–1^ s^–1^ (on–off ratio 2 × 10^7^) and an electron mobility of 0.05 cm^2^ V^–1^ s^–1^ (on–off ratio 5 × 10^5^). The FETs obtained from **PNDIC8TEG-TVT**-sorted s-SWCNT exhibit ambipolar characteristics with a hole mobility of 2.31 cm^2^ V^–1^ s^–1^ (on–off ratio 2 × 10^6^) and an electron mobility of 0.38 cm^2^ V^–1^ s^–1^(on–off ratio 2 × 10^6^).

**Figure 6:**
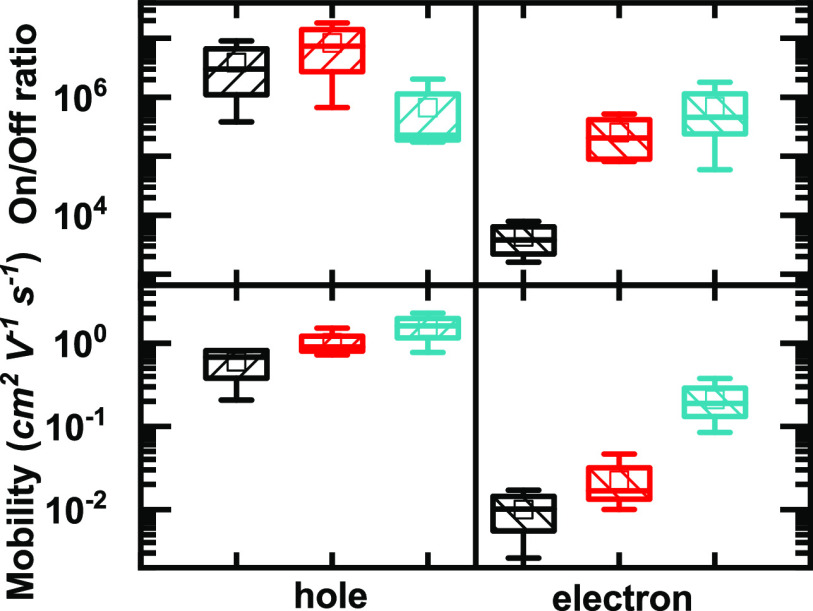
Figures of merit of the FETs fabricated from HiPCO:**PNDITEG-AH** (black), HiPCO:**PNDITEG-TVT** (red), and HiPCO:**PNDIC8TEG-TVT** (turquoise) inks.

A summary of the FET performance distribution in devices fabricated with these enriched polymer-sorted s-SWCNT inks is reported in Figure 6. It is clear that HiPCO:**PNDITEG-AH**-based FETs demonstrate the lowest performance as compared with that of HiPCO:**PNDITEG-TVT**- and HiPCO:**PNDIC8TEG-TVT**-based FETs in both hole and electron mobility, despite the fact that **PNDITEG-AH** exhibits the highest dispersion capability as indicated by the absorption spectra. In contrast with the absorption spectra, the FET data give a clear indication that there are no metallic tubes present in the devices. All transistors were functional and the hole on–off ratios is in the order of 10^6^ or above.

### Energy Level Alignment and FET Hysteresis

**Figure 7:**
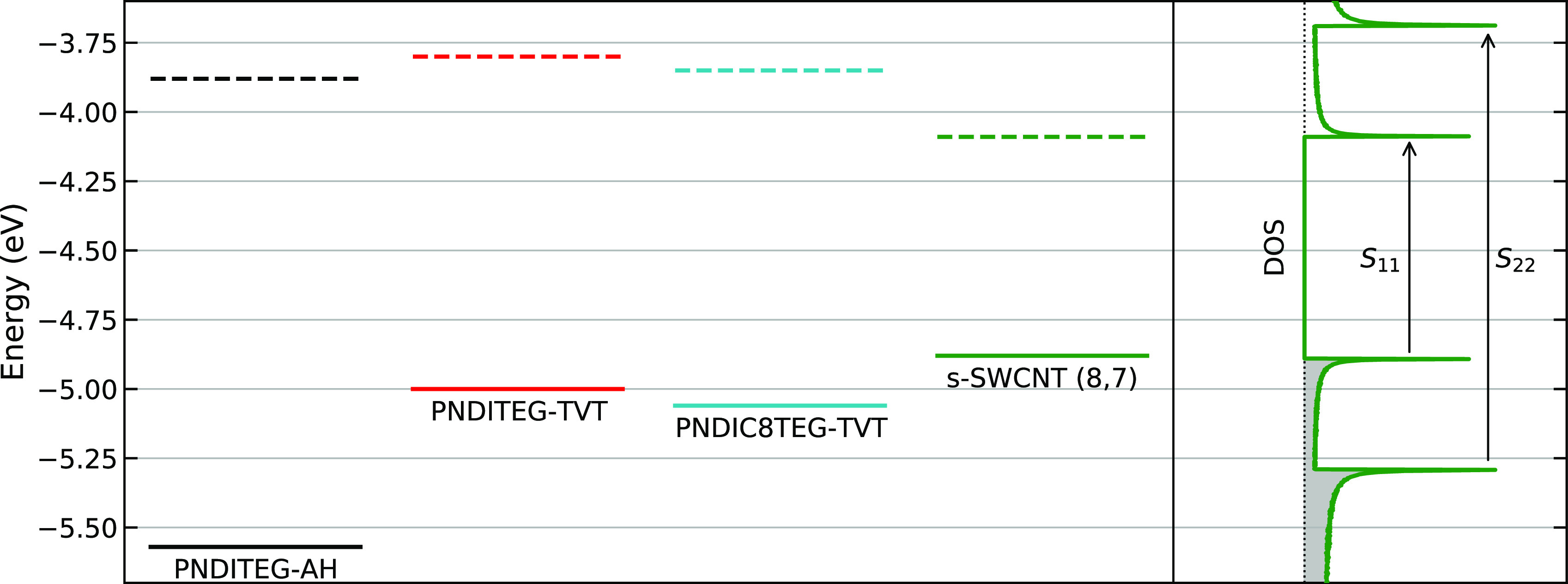
Energy levels (solid for HOMO, dashed for LUMO) of **PNDITEG-AH**, **PNDITEG-TVT**, and **PNDIC8TEG-TVT** compared with s-SWCNT (8,7). On the diagram on the right is displayed the density of states for the (8,7) s-SWCNT schematically represented. The s-SWCNT data for HOMO and LUMO are taken from ref (59).

As described above, the FET performances can be affected by the energy levels and mobility of polymers. Figure 7 shows the energy level alignment of the three investigated polymers and the (8,7) s-SWCNT. Compared with **PNDITEG-AH**, the higher HOMO level of vinylene-containing polymers **PNDITEG-TVT** and **PNDIC8TEG-TVT** result in an improved energy alignment with the HOMO levels of the s-SWCNT. Therefore, the energy barrier for intertube hole transport in a network for NDI-TVT based polymer-wrapped s-SWCNT is lower than that of NDI-AH based polymer-wrapped s-SWCNT. In the case of electron transport, these three polymers show energy alignment similar to that of (8,7) s-SWCNT. However, as compared with previously reported NDI-TVT-based polymers, the electron mobility of **PNDITEG-AH** is expected to be lower.^45,47,58^ This can be explained by the more twisted backbone of **PNDITEG-AH**. This lower electron mobility within the polymer results in a higher energy barrier for electron transport from one s-SWCNT to another, reducing overall FET electron mobilities.

The devices made from the HiPCO:**PNDIC8TEG-TVT** ink display higher mobilities as compared to **PNDITEG-TVT**, for both hole and electron conduction. Combining these data with the absorption spectra, we suspect this improvement is likely due to a higher polymer dispersion efficiency, i.e., more s-SWCNT are selected with respect to the remaining free polymer in the ink after purification. The lower amount of free polymer significantly reduces the barrier between the s-SWCNT for intratube charge transport, resulting in the higher FET performances seen for **PNDIC8TEG-TVT** as compared with that of **PNDITEG-TVT**. Other data supporting this hypothesis include that FET devices fabricated immediately after selection (before enrichment) using the **PNDITEG-TVT** s-SWCNT ink show 100 times higher hole mobility than that of **PNDIC8TEG-TVT**. After enrichment, the excess polymer is removed, and the **PNDIC8TEG-TVT** solution is still superior over **PNDITEG-TVT**. Additionally, **PNDIC8TEG-TVT**-based FET displays more ambipolar electrical characteristics than **PNDITEG-TVT**-based FETs. The electron mobility of the **PNDIC8TEG-TVT**-based FET is only 1 order of magnitude lower in hole mobility. Interestingly, the increase of ambipolarity in this sample slightly degrades the hole on–off ratio of the device, giving values above 10^5^. This is also reflected by statistics over all devices (Figure 6 and Table S3). We note that ambipolarity in a FET for many applications is undesirable; however, polarity tuning through the use of dopants has previously been demonstrated as a potential solution.^5^

To further help clarify the role of the wrapping polymer, the FET hysteresis is examined. The hysteresis was quantified by taking the difference in threshold voltage (*V*_th_) between forward and backward sweep, as obtained during the transfer characteristics measurements (summarized in Table S3). Interestingly, the hysteresis for holes in HiPCO:**PNDITEG-AH** is significantly larger than the value as obtained for HiPCO:**PNDITEG-TVT** or HiPCO:**PNDIC8TEG-TVT**. This can be explained by the higher energetic barrier of **PNDITEG-AH**, which enhances the possibility of trapping. In contrast, the hysteresis for electron transport is slightly lower for HiPCO:**PNDITEG-AH**, although similar to the one of the other two polymers. Indeed, the LUMO of this particular polymer lies slightly closer to that of the s-SWCNT, as compared with **PNDITEG-TVT** and **PNDIC8TEG-TVT** (Figure 7). These data support our hypothesis that the energy levels of **PNDITEG-TVT** and **PNDIC8TEG-TVT** are better aligned with the s-SWCNT, reducing the energy barrier for holes and improving charge transport.

Overall, the HiPCO:SWCNT dispersions (obtained with **PNDITEG-AH**, **PNDITEG-TVT**, and **PNDIC8TEG-TVT**) are of very good quality and demonstrate that small variations of the chemical structure of the wrapping polymer can have a large influence on the transport properties of the inks.

## Conclusion

We have successfully demonstrated that two newly synthesized naphthalene diimide based narrow-band-gap conjugated polymers are able to wrap and select semiconducting SWCNT. Compared to the conjugated polymers with naphthalene diimide and head-to-head bithiophene units (**PNDITEG-AH**), we found that inserting a small conjugated vinyl group into the head-to-head bithiophene substantially increases backbone planarity and lowers the band gap of the corresponding conjugated polymers (**PNDITEG-TVT** and **PNDIC8TEG-TVT**) without sacrificing the wrapping ability significantly. Furthermore, usage of an alkyl chain spacer between conjugated backbone and oligomer glycol ether side chain increases the flexibility and solubility of **PNDIC8TEG-TVT**, which leads to efficient removal of excess polymer from the s-SWCNT ink during the enrichment step. Field-effect transistors fabricated from **PNDITEG-AH**-, **PNDITEG-TVT**-, and **PNDIC8TEG-TVT**-wrapped s-SWCNT were fabricated and used as a tool to evaluate the influence of the backbone on the device performance. The hole transport of devices fabricated with **PNDITEG-AH**, **PNDITEG-TVT**, and **PNDIC8TEG-TVT** selected s-SWCNT increased gradually from twisted polymers **PNDITEG-AH** to planar polymers **PNDITEG-TVT** and **PNDIC8TEG-TVT**. The higher performance of **PNDITEG-TVT** and **PNDIC8TEG-TVT** originate from their improved HOMO level alignment with nanotubes, as compared with that of **PNDITEG-AH**. Our work provides insight into the fundamental understanding of the relationship between the molecular structure of the polymer and corresponding dispersion capability of SWCNT and physical properties of the final inks.

## Experimental Section

### Preparation and Characterization of s-SWCNT Dispersions

HiPCO SWCNT (0.8 nm 1.2 nm) inks and devices were prepared and characterized as described in our previous work.^60^ FET performances (per ink) are based on multiple transistors residing on a chip, for multiple chips. A detailed description of the characterization of the inks and fabrication of the transistors is available in the Supporting Information.

### DFT Calculations

DFT calculations were performed using Gaussian 16 program with B3LYP functional and 6-311G(d) basis set. We choose one repeat unit of each polymer as the model molecule in our simulation to reduce the calculation time. These model molecules were first optimized at gas phase. Then we used the optimized geometry as the starting point for further single point energy calculations.

## Supporting Information Available

Detailed description of the synthesis and characterization of the materials; NMR and FT-IR spectra; Maldi-TOF-MS spectra; TGA and DSC curves; DFT calculation; preparation and characterization of s-SWCNT dispersions; UV–vis–NIR spectra; transistor device fabrication and measurement; AFM images and other data (PDF)
