# Supplementary material for: Efficient Selective Sorting of Semiconducting Carbon Nanotubes Using Ultra-Narrow-Band-Gap Polymers
Source: ACS Appl Mater Interfaces. 2022 Aug 9;14(33):38056–66. doi: 10.1021/acsami.2c07158 (PMC9412849; doi:10.1021/acsami.2c07158)
Supplement: Supplementary file 1 — am2c07158_si_001.pdf [file am2c07158_si_001.pdf]

Supporting Information: Efficient Selective Sorting of Semiconducting Carbon Nanotubes Using Ultranarrow-Bandgap Polymers

Wytse Talsma,[†,‡] Gang Ye,[*,¶,§,†,‖,‡] Yuru Liu,[§,†] Herman Duim,[†] Sietske Dijkstra,[†] Karolina Tran,[†] Junle Qu,[¶] Jun Song,[¶] Ryan C. Chiechi,[*,§,⊥] and Maria Antonietta Loi[*,†]

†Zernike Institute for Advanced Materials, University of Groningen, Nijenborgh 4, 9747 AG Groningen, The Netherlands

‡These authors contributed equally to this work.

¶Center for Biomedical Optics and Photonics (CBOP) & College of Physics and Optoelectronic Engineering, Key Laboratory of Optoelectronic Devices and Systems, Shenzhen University, Shenzhen 518060, P. R. China

§Stratingh Institute for Chemistry, University of Groningen, Nijenborgh 4, 9747 AG Groningen, The Netherlands

‖Present address: State Key Laboratory of Polymer Physics and Chemistry, Changchun Institute of Applied Chemistry, Chinese Academyof Sciences, Changchun, 130022 Jilin, P. R. China

⊥Department of Chemistry & Carbon Electronics Cluster, North Carolina State University, Raleigh, North Carolina 27695-8204, United States

E-mail: g.ye0612@ciac.ac.cn; ryan.chiechi@ncsu.edu; m.a.loi@rug.nl

Contents

1 General Experimental S-3

2 Materials and Synthesis S-5

3 ^{1}HNMR of Polymers S-19

4 Fourier transform infrared spectroscopy (FT-IR) S-21

5 Gel Permeation Chromatography (GPC) S-23

6 Matrix-assisted laser desorption/ionization time-of-flight mass (MALDI-TOF-MS) S-25

7 Thermal Properties S-28

8 Optical Properties S-29

9 Density Functional Theory Calculation S-30

10 Preparation and characterization of s-SWCNT dispersions S-35

11 Optical Characterization of the s-SWCNT Dispersion S-35

12 FET transistor fabrication and electrical characterization S-37

13 Atomic Force Microscopy (AFM) image of FET device S-38

References S-40

1 General Experimental

^{1}HNMR and ^{13}CNMR were performed on a Varian Unity Plus (400 MHz) instrument at 25 °C, using tetramethylsilane (TMS) as an internal standard. NMR shifts are reported in ppm, relative to the residual protonated solvent signals of $CDCl_3$ (σ=7.26 ppm) or at the carbon absorption in $CDCl_3$ (σ = 77.23 ppm). Multiplicities are denoted as: singlet (s), doublet (d), triplet (t) and multiplet (m). High Resolution Mass Spectroscopy (HRMS) was performed on a JEOL JMS 600 spectrometer. FT-IR spectra were recorded on a Nicolet Nexus FT-IR fitted with a Thermo Scientific Smart iTR sampler. GPC measurements were done on a Waters1525 GPC/SEC system at 35 °C *vs* polystyrene standards using trichloroform as eluent. Thermal properties of the polymers were determined on a TA Instruments DSC Q20 and a TGA Q50. DSC measurements were executed with two heating-cooling cycles with a scan rate of 10 °C min^{-1}, and from each scan, the second heating cycle was selected. TGA measurements were done from 20 °C to 800 °C with a heating rate of 20 °C min^{-1}. Cyclic voltammetry (CV) was carried out with an Autolab PGSTAT100 potentiostat in a three-electrode configuration where the working electrode was a glassy carbon electrode, the counter electrode was a platinum wire, and the pseudo-reference was a Ag/AgCl wire that was calibrated against ferrocene (Fc/Fc^+). The energy levels were calculated using the well-known empirical relations as described in literature.[S1,S2] Cyclic voltammograms for NDI-Based polymers film deposited on the glass carbon working electrode in CH_3CN solution containing Bu_4NPF_6 (0.1 mol l^{-1}) electrolyte at a scanning rate of 100 mV s^{-1}. Density functional theory (DFT) calculations were performed using Gaussian 16.[S3] Target model molecules were first optimized at gas phase and then single point energies were calculated. Both calculations were performed with B3LYP functional and 6-311G(d) basis set.

Non-resonant Raman scattering was performed on the shelved inks using a confocal Raman microscope (invia, Renishaw) and by using an integrated 532 nm solid state laser. Scattered light was sent through a 15 cm^{-1} low energy edge filter, dispersed with a 1800 l mm^{-1} grating and detected using a silicon CCD panel. The data is corrected for the baseline

by the equipment, and normalized (max./min.) afterwards.

2 Materials and Synthesis

Synthesis and Characterization

Reagents: All reagents and solvents were commercial and were used as received. 5,10-Dibromoisochromeno[6,5,4-def]isochromene-1,3,6,8(3aH,8aH)-tetraone was purchased from SunaTech Inc.

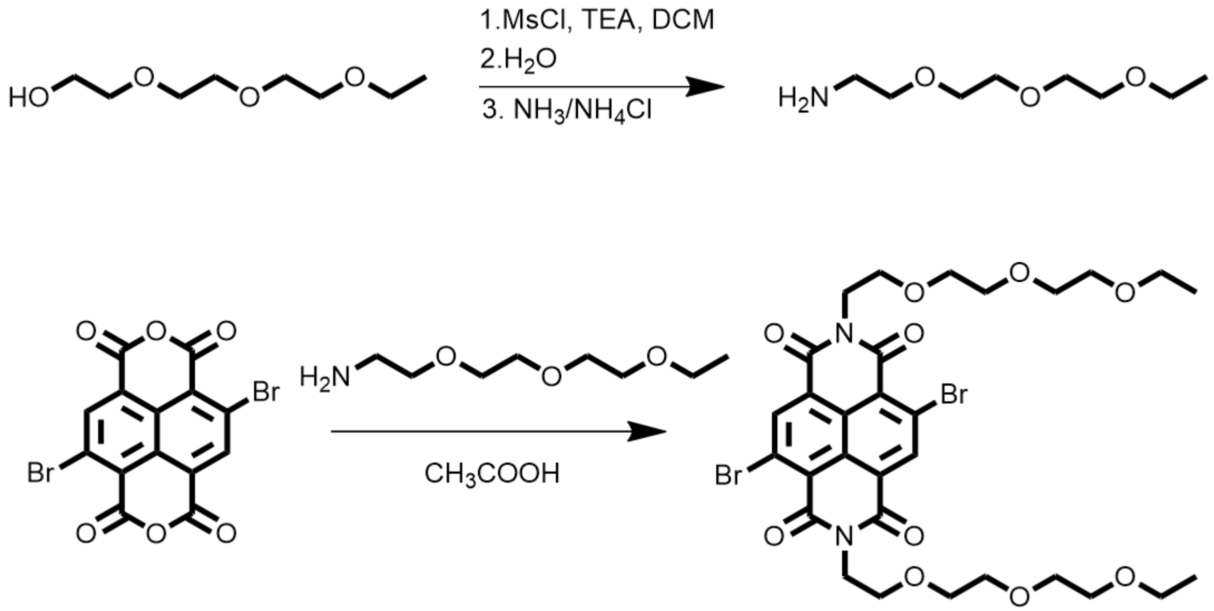

Figure S1: Synthetic route to the naphthalenediimide based monomer NDITEG. (Adapted from our previous work[S4])

4,9-dibromo-2,7-bis(2-(2-(2-ethoxyethoxy)ethoxy)ethyl)benzo[lmn][3,8]phenanthroline-1,3,6,8(2H,7H)-tetraone (NDITEG) was synthesized according to literature procedures.[S5–S7] NDITEG: ^{1}HNMR (400 MHz, $CDCl_3$): δ : 8.99 (s, 2H), 4.47 (t, J = 5.6 Hz, 4H), 3.85 (t, J = 6.0 Hz 4H), 3.73-3.67 (m, 4H), 3.64-3.56 (m, 8H), 3.54-3.43 (m,8H), 1.17 (t, J = 6.8 Hz 6H). ^{13}CNMR (100 MHz, $CDCl_3$) δ: 160.72, 160.62, 138.92, 128.27, 127.65, 125.24, 124.01, 70.60, 70.56, 70.06, 69.71, 67.52, 66.55, 39.99, 15.10.

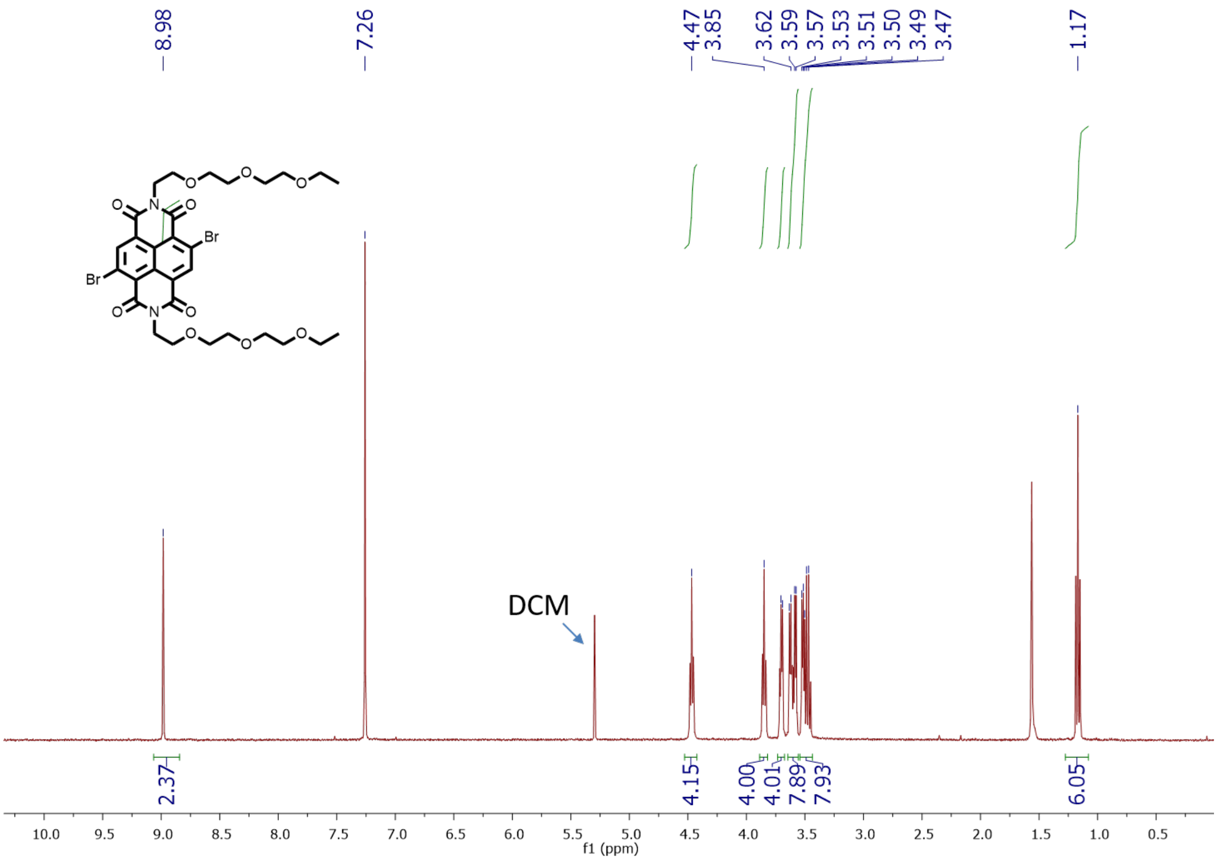

Figure S2: ^{1}HNMR spectra of NDITEG (Adapted from our previous work[S4])

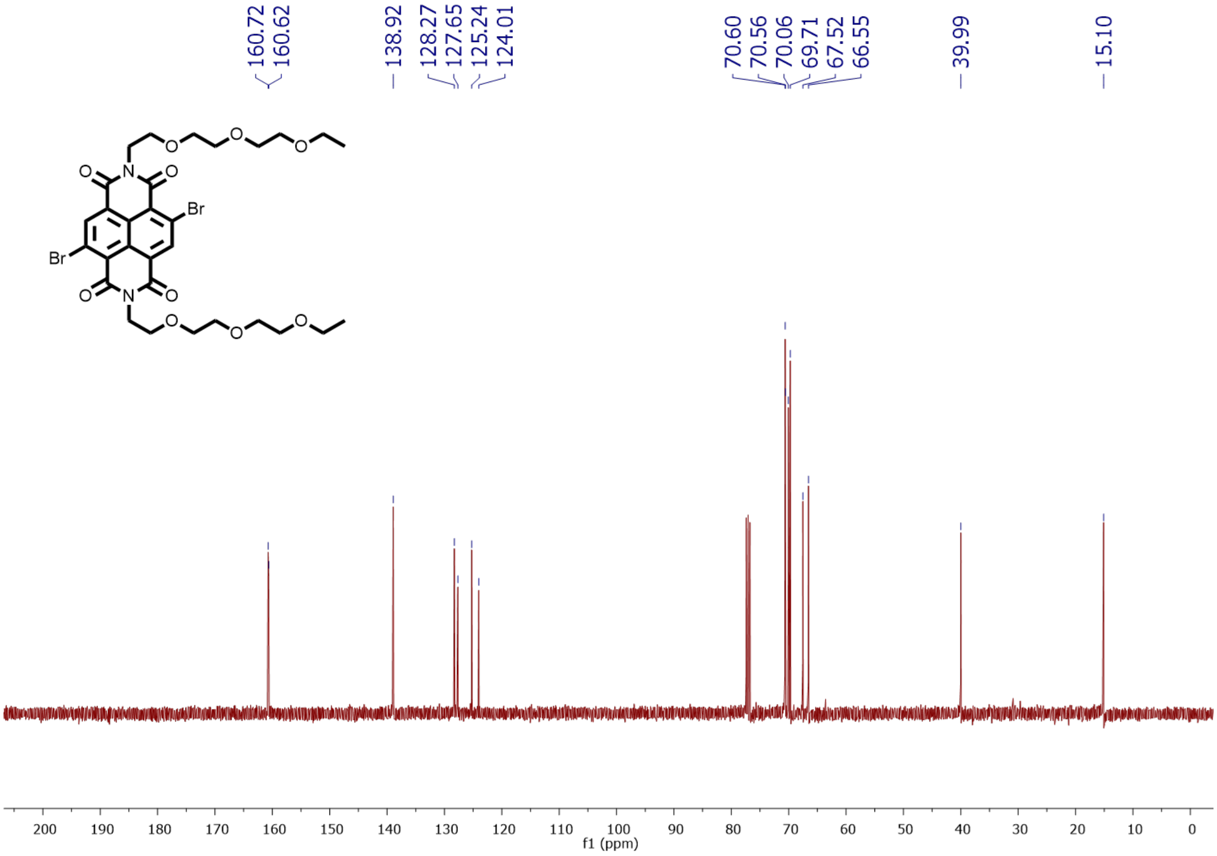

Figure S3: ^{13}CNMR spectra of NDITEG (Adapted from our previous work[S4])

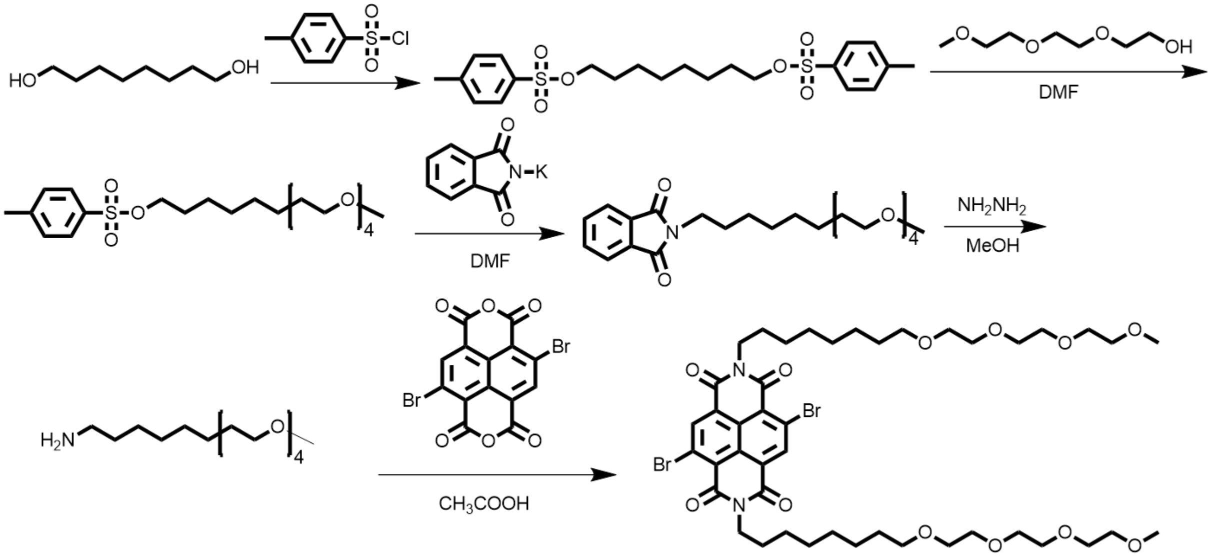

Figure S4: Synthetic route to the naphthalenediimide based monomer NDIC8TEG

4,9-dibromo-2,7-di(2,5,8,11-tetraoxanonadecan-19-yl)benzo[lmn][3,8]phenanthroline-1,3,6,8(2H,7H)-

tetraone (NDIC8TEG) was synthesized according to literature procedures.[S8]

NDIC8TEG: ^{1}HNMR (400 MHz, $CDCl_3$): δ : 8.99 (s, 2H), 4.18 (t, J = 5.6 Hz, 4H), 3.70-3.60 (m, 16H), 3.60-3.50 (m, 8H), 3.44 (t, 4H), 3.37 (s, 6H), 1.80-1.68 (m, 4H), 1.65-1.51 (m, 4H), 1.47-1.27 (m, 16H). ^{13}CNMR (100 MHz, $CDCl_3$) δ: 163.39, 163.37, 141.72, 130.98, 130.37, 127.98, 126.72, 74.58, 74.12, 73.26, 73.23, 73.17, 72.70, 61.70, 44.23, 32.24, 32.00, 31.88, 30.52, 29.67, 28.67. HRMS(ESI) calcd. for $C_{44}H_{65}Br_2N_2O_{12}$ $[M+H]^+$: 973.28783, found: 973.28811; calcd. for $C_{44}H_{65}Br_2N_2O_{12}$ $[M+NH4]^+$: 990.31438, found: 990.31475.

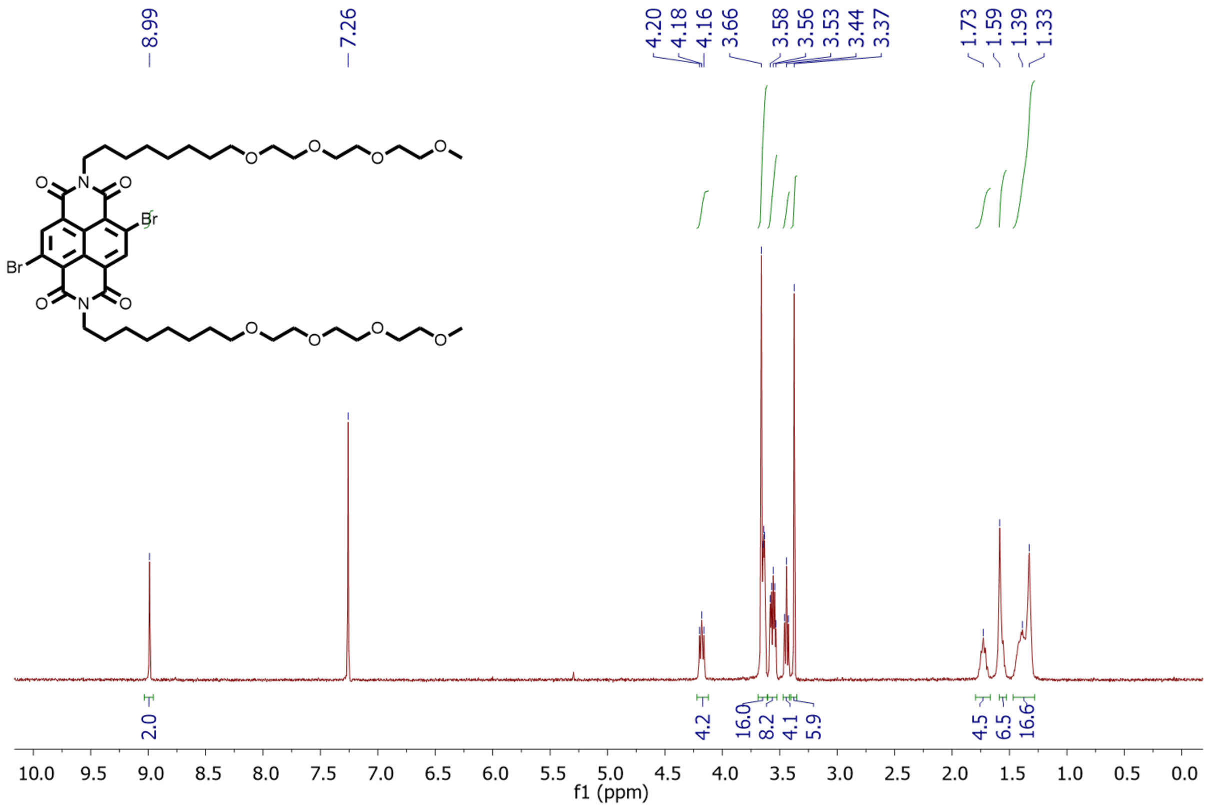

Figure S5: The ^{1}HNMR spectra of NDIC8TEG, (Adapted from our previous work[S9]

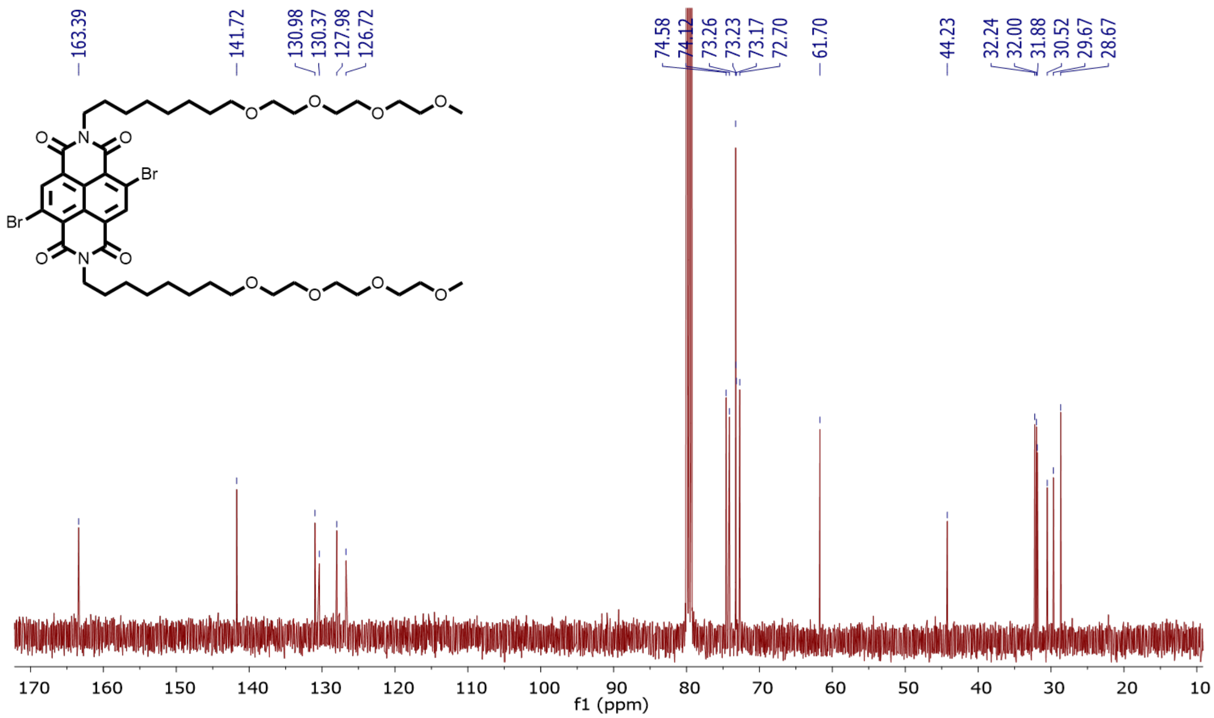

Figure S6: The ^{13}CNMR spectra of NDIC8TEG, (Adapted from our previous work[S9]

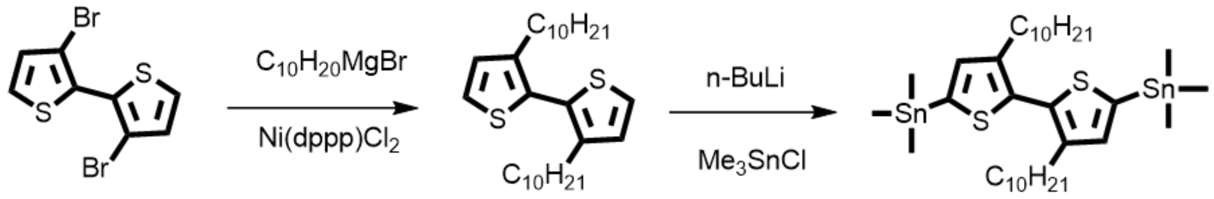

Figure S7: Synthetic route to the head to head bithiophene based monomer.

(3,3'-didecyl-[2,2'-bithiophene]-5,5'-diyl)bis(trimethylstannane) (AH) was synthesized according to literature procedures.[S4,S5]

AH: ^{1}HNMR (400 MHz, $CDCl_3$): δ : 7.02 (s, 2H), 2.51 (t, J = 8.0 Hz, 4H), 1.57 (m, 4H), 1.40-1.17 (br, 28H), 0.88 (t, J = 6.8 Hz, 6H), 0.363 (s, 18H).

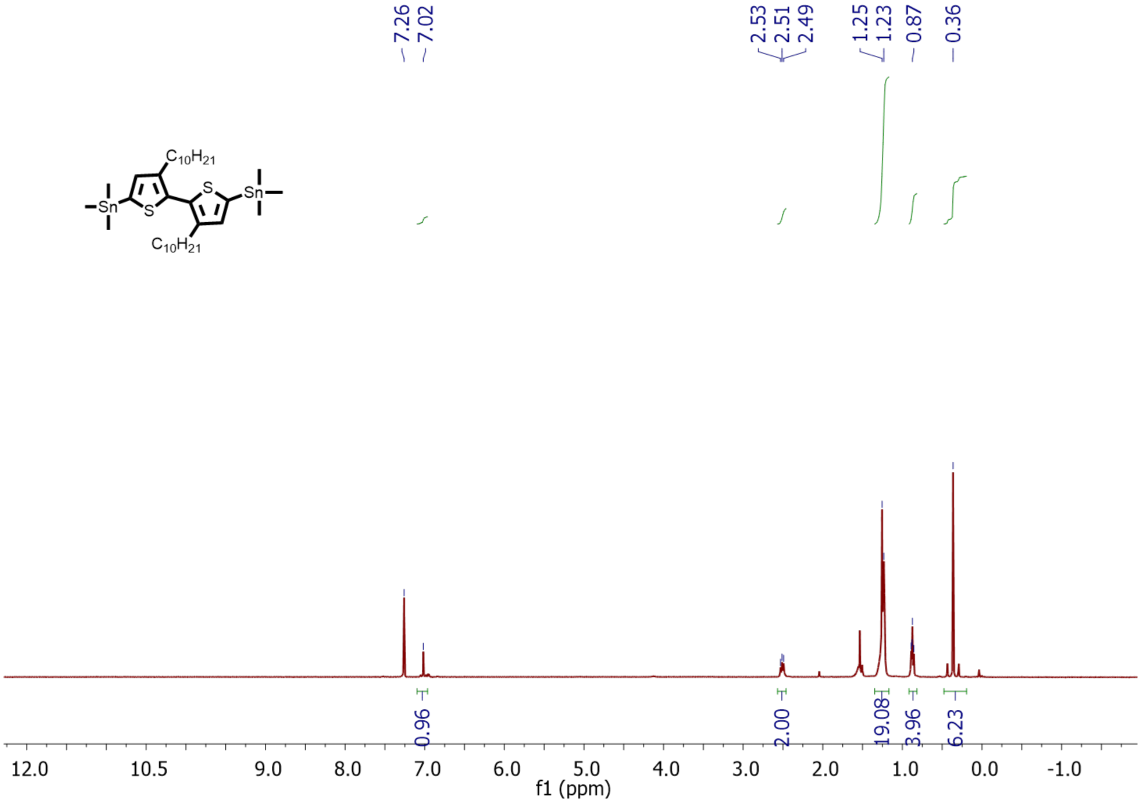

Figure S8: The ^{1}HNMR spectra of (3,3'-didecyl-[2,2'-bithiophene]-5,5'-diyl)bis(trimethylstannane) (Adapted from our previous work[S4])

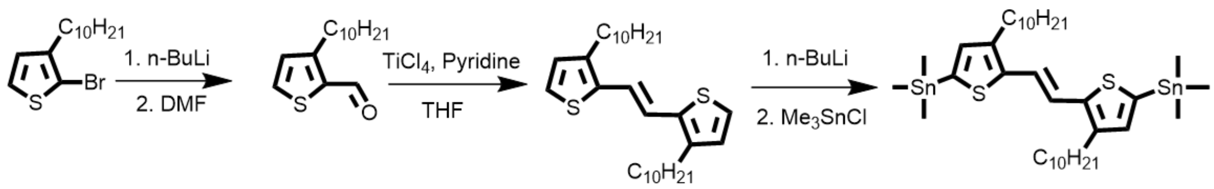

Figure S9: Synthetic route to the thienylennevinylene based monomer.

3-decylthiophene-2-carbaldehyde[S10]

To a dry three-neck flask was added 2-bromo-3-decylthiophene (3000 mg, 10 mmol and anhydrous THF (40 mL) under an atmosphere of N_2, cooled to -78 °C. n-butyllithium (6.51 mL, 10.42 mmol, 1.6 M in hexane,) was added drop-wise. The solution was stirred for 1 hours in the cold bath at -78 °C before dimethylformamide (DMF) (2 mL, 26 mmol) was added. After the mixture was stirred at room temperature for 12 hours, (100 mL water was added. The mixture was extracted with n-hexane (2 mL). The organic phase was washed with brine, and dried over anhydrous Na_2SO_4. The solvent was then evaporated by rotary evaporator under reduced pressure. The crude residue was purified by column chromatography to give pure target product (2.0 g, 80 %). ^{1}HNMR (400 MHz, $CDCl_3$): δ : 10.04 (s, 1H), 7.64 (d, J = 4.8Hz 1H), 7.01 (d, J = 4.8Hz, 1H), 2.96 (t, J = 8.0 Hz, 2H), 1.67 (m, 2H), 1.40-1.17 (br, 14H), 0.88 (t, J = 6.8 Hz, 3H). ^{13}CNMR (100 MHz, $CDCl_3$) δ: 182.23, 152.87, 137.62, 134.36, 130.66, 31.85, 31.42, 29.54, 29.49, 29.34, 29.27, 28.48, 22.65, 14.08.

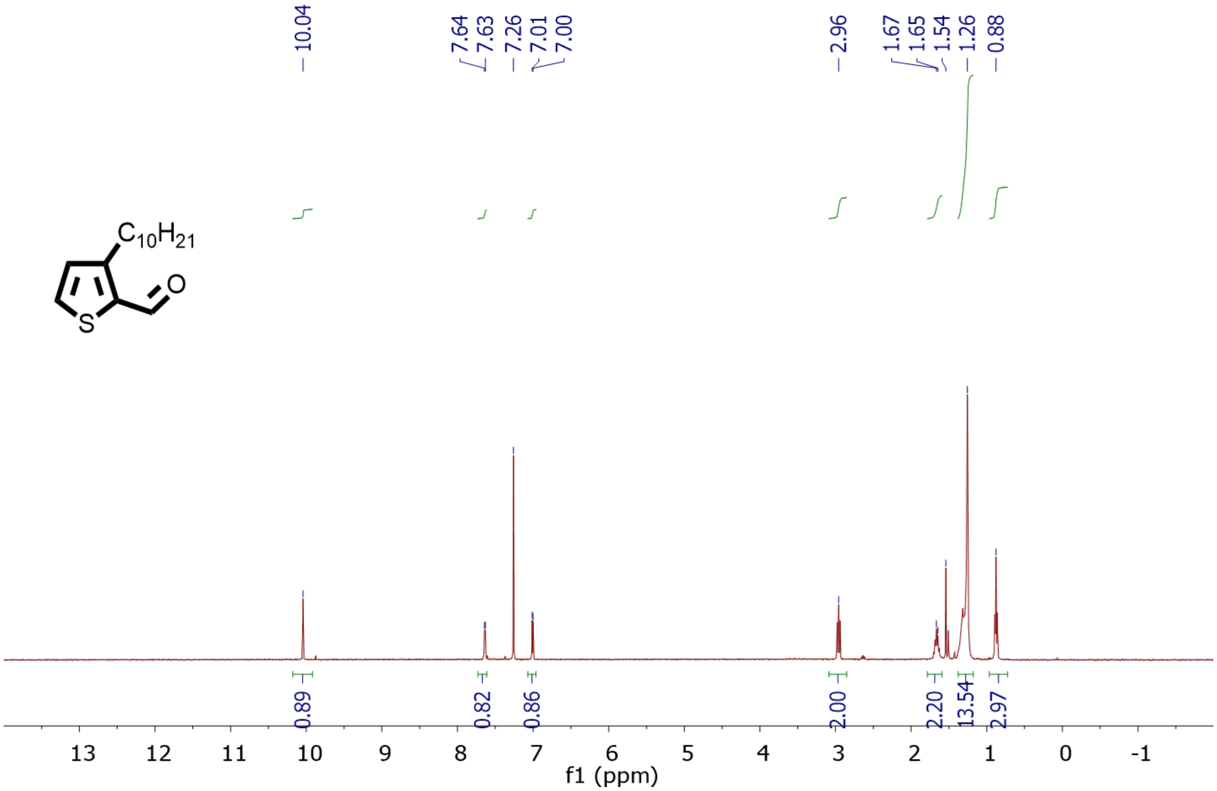

Figure S10: The ¹HNMR spectra of 3-decylthiophene-2-carbaldehyde

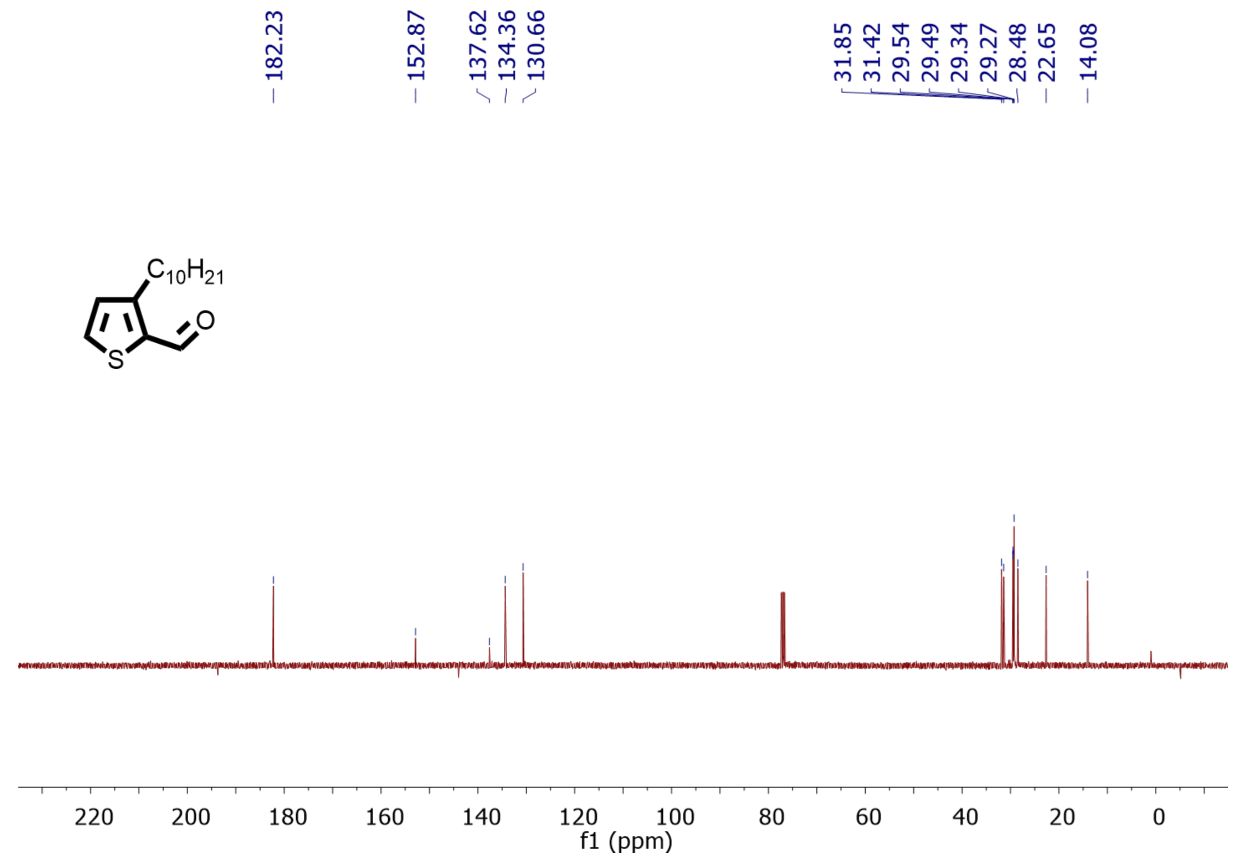

Figure S11: The ^{1}HNMR spectra of 3-decylthiophene-2-carbaldehyde

(E)-1,2-bis(3-decylthiophen-2-yl)ethene[S11]

To a dry three-neck flask was added anhydrous THF (15 mL). $TiCl_4$ (0.22 mL) and Zn powder (0.4 g) were added at 0°C. The mixture was stirred at 0°C for 15 mins, and then refluxed at 70°C for 1 hr. The color changed from green into dark blue. Then prepared 3-decylthiophene-2-carbaldehyde (500 mg, 2 mmol) and pyridine (0.16 mL, 2 mmol) was then added into the flask and reflux for 2 hours. After the solution was cooled to room temperature, water (100 mL) was added to quenched the reaction. The mixture was extracted with n-hexane (100 mL). The organic phase was washed with brine, and dried over anhydrous Na_2SO_4. The solvent was then evaporated by rotary evaporator under reduced pressure. The crude residue was purified by column chromatography (SiO_2, hexane). The pure product was obtained by recrystallization from isopropyl alcohol. ^{1}HNMR (400 MHz, $CDCl_3$): δ : 7.07 (d, J = 5.2 Hz, 2H), 6.99 (s, 2H), 6.85 (d, J = 5.2Hz, 2H), 2.65 (t, J = 7.6 Hz, 4H), 1.59 (m, 4H),

1.40-1.17 (br, 28H), 0.87 (t, J = 5.8Hz, 6H). ^{13}CNMR (100 MHz, $CDCl_3$) δ: 140.70, 136.23, 129.80, 122.47, 119.41, 31.89, 30.90, 29.61, 29.58, 29.45, 29.34, 29.32, 28.41, 22.67, 14.10. HRMS(ESI) was calculated for $C_{30}H_{49}S_2$ $[M+H]^+$ 473.32601 and found to be 473.32702.

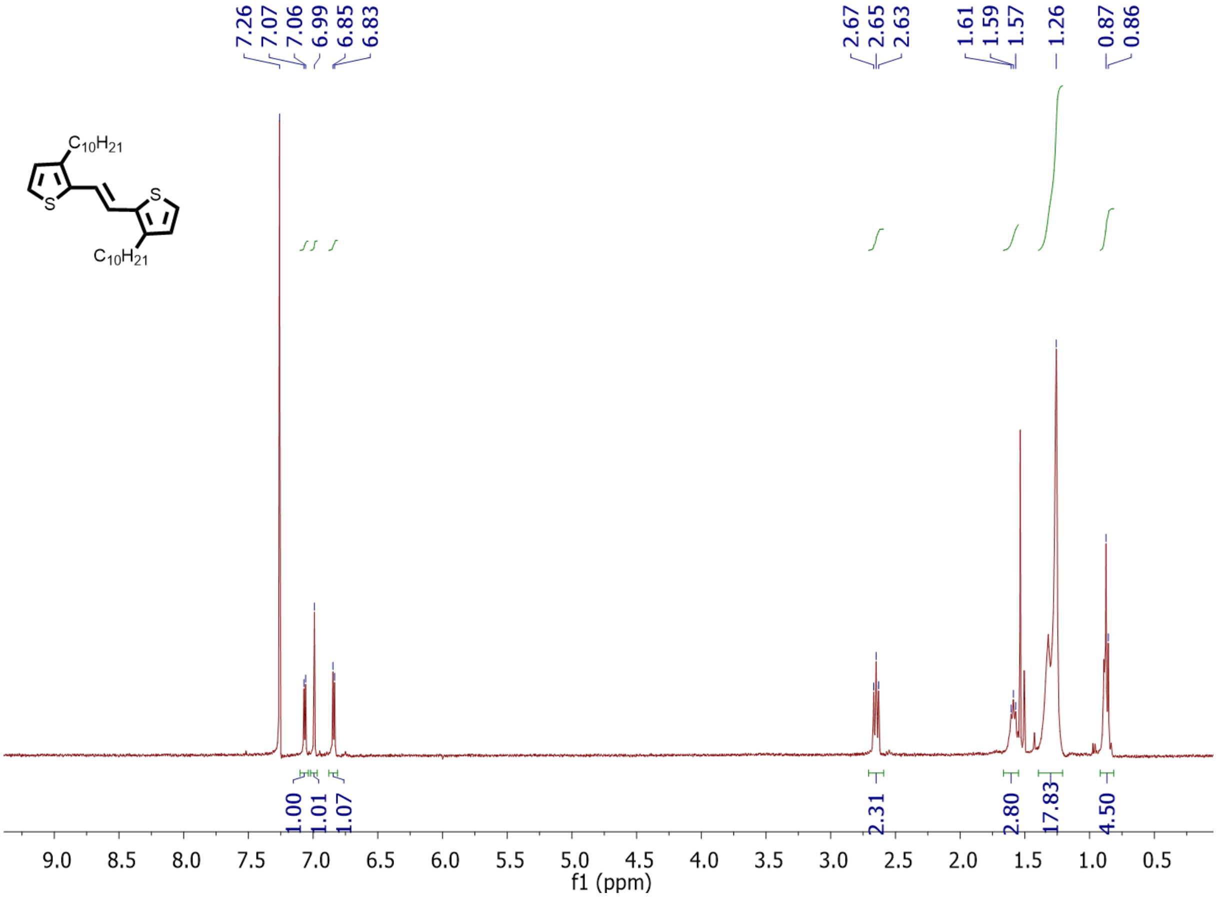

Figure S12: The ^{1}HNMR spectra of (E)-1,2-bis(3-decylthiophen-2-yl)ethene

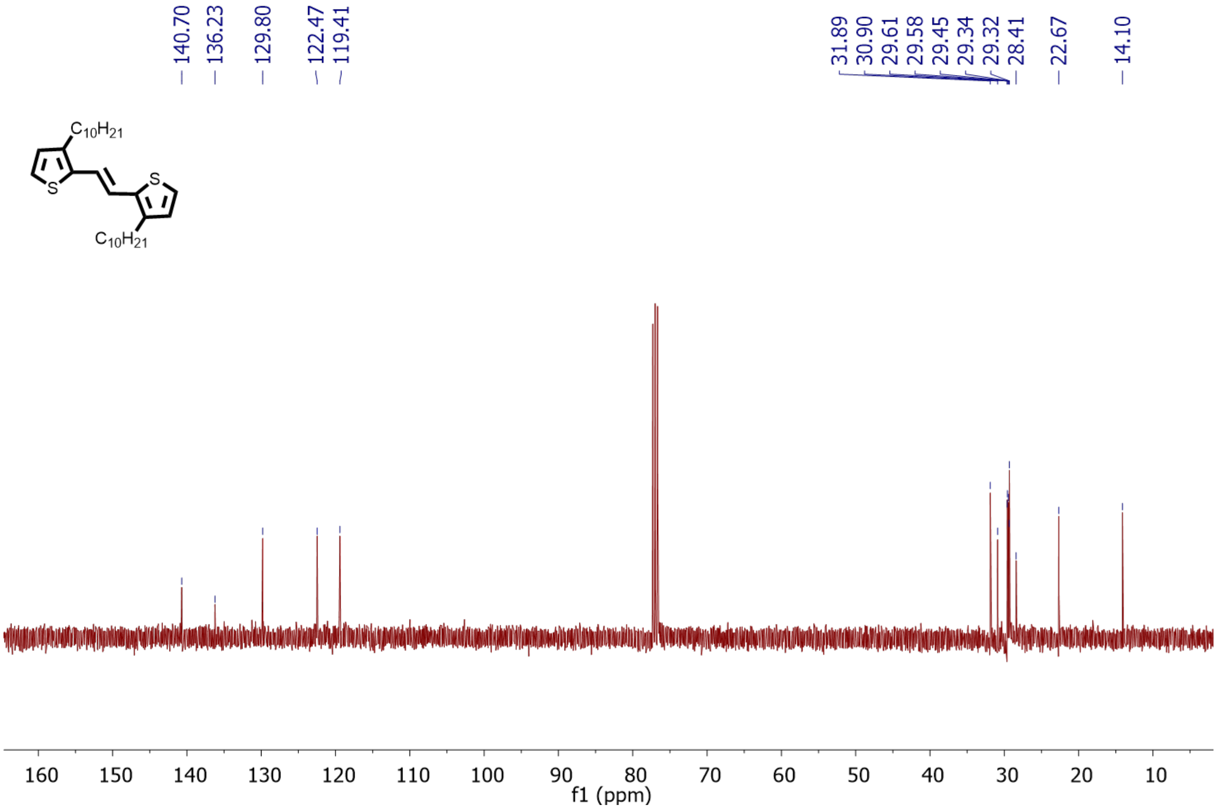

Figure S13: The ^{1}HNMR spectra of (E)-1,2-bis(3-decylthiophen-2-yl)ethene

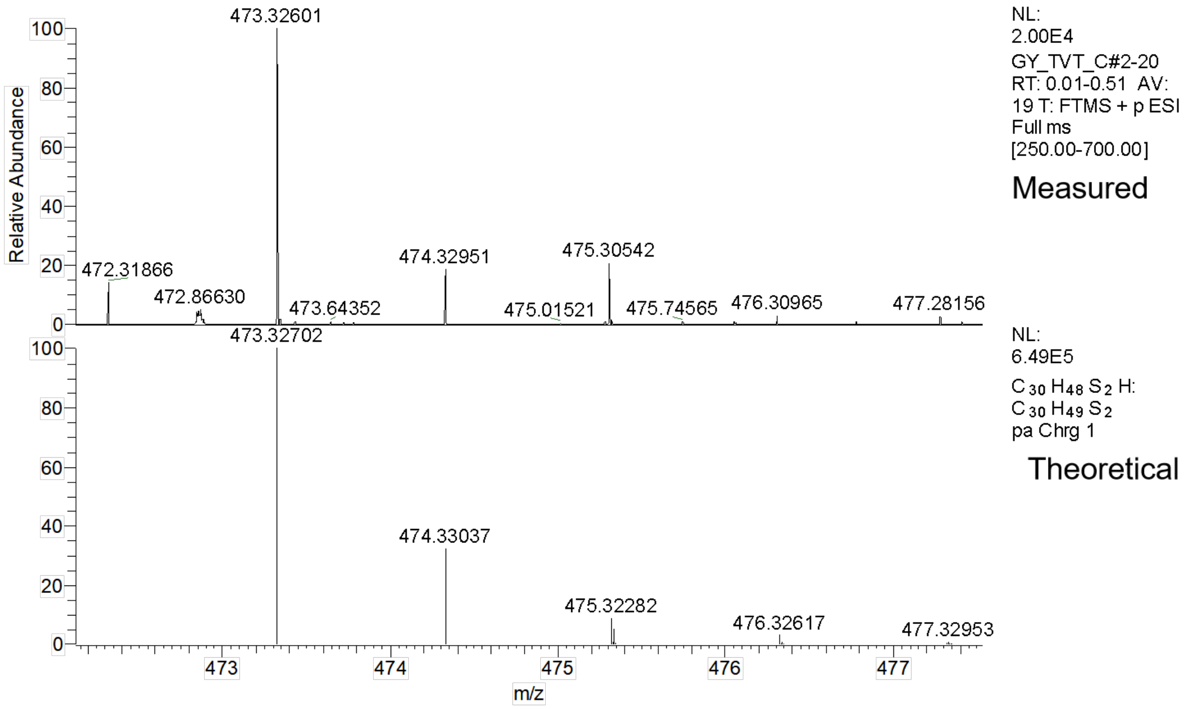

Figure S14: The HRMS spectra of (E)-1,2-bis(3-decylthiophen-2-yl)ethene

(E)-1,2-bis(3-decyl-5-(trimethylstannyl)thiophen-2-yl)ethene [S12,S13]

To a dry three-neck flask was added (E)-1,2-bis(3-decylthiophen-2-yl)ethene (270 mg, 0.6 mmol) and 10 mL of anhydrous THF under an atmosphere of N_2, and cooled to -10 °C and n-butyllithium (1.32 mmol, 0.85 mL, 1.6 M in hexane) was added drop-wise. The solution was stirred for 1 hours at -10 °C. Then, the mixture was warmed to room temperature and stirred at room temperature for 1 hour, and cooled again to -55 °C. Then, trimethyltin chloride (3.6 mL, 3.6 mmol, 1.0 M in THF) was added to the resulting reaction mixture, which was stirred for 30 min then warmed again to room temperature. After that, the solution was stirred at room temperature overnight. Water was added to quench the reaction, and the solution was extracted with n-hexane. The organic phase was dried over Na_2SO_4 and the solvent was removed by rotary evaporation. The pure target product was recrystallized from isopropyl alcohol to give thienylennevinylene monomer (400 mg, yield: 88 %). ^{1}HNMR (400 MHz, $CDCl_3$): δ : 6.99 (s, 2H), 6.91 (s, 2H), 2.65 (t, J = 7.6 Hz, 4H), 1.58 (m, 4H), 1.40-1.17 (br, 28H), 0.87 (t, J = 7.2 Hz, 6H), 0.36 (s, 18H).

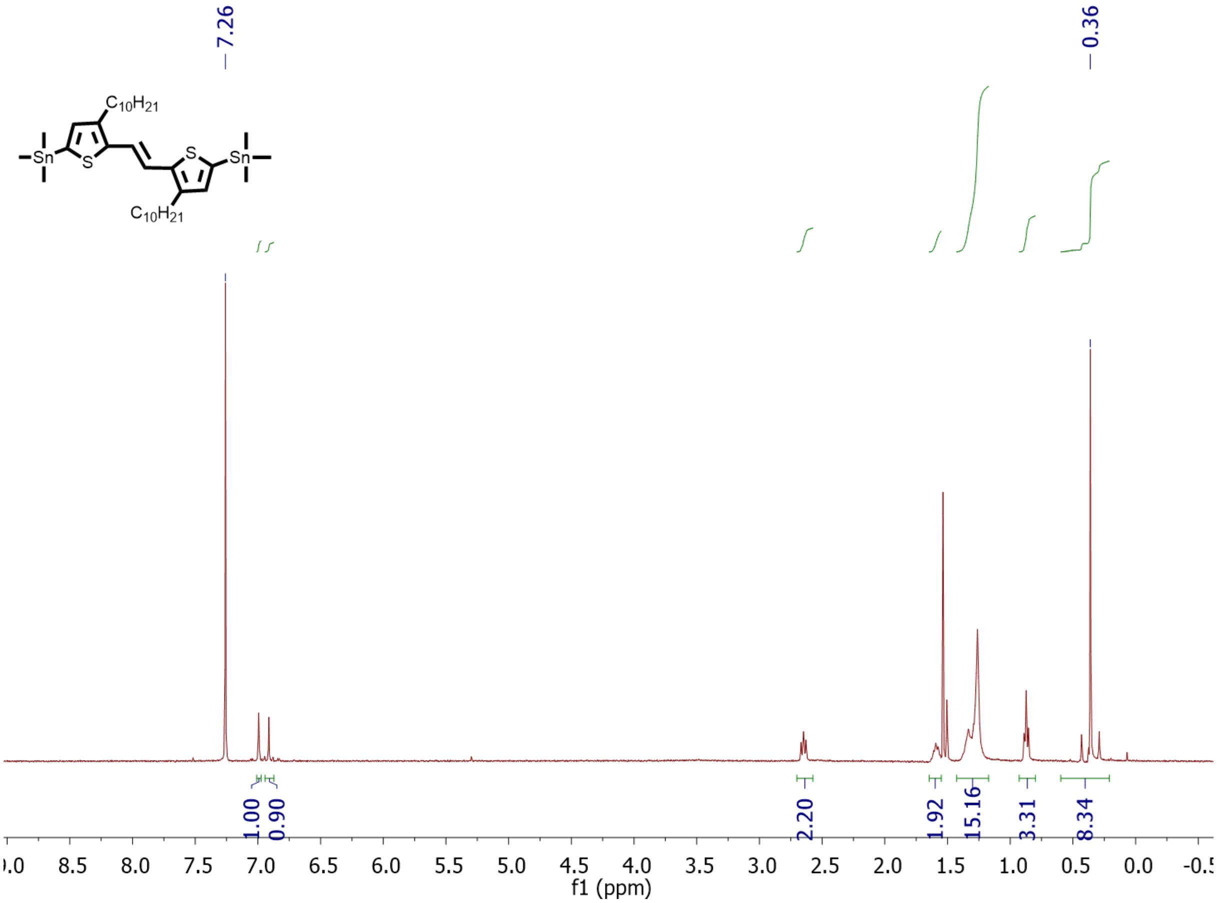

Figure S15: The ^{1}HNMR spectra of (E)-1,2-bis(3-decyl-5-(trimethylstannyl)thiophen-2-yl)ethene

General synthetic procedures for the naphthalenediimide (NDI) based polymer

To a dry three-neck flask, NDI based monomer (0.1 mmol) and thiophene based monomer (0.1 mmol) were added under argon followed by tris(dibenzylideneacetone) dipalladium $Pd_2(dba)_3$ (8 mg)and tri(o-tolyl)phosphine $P(o\text{-}tolyl)_3$(12 mg). The flask and its contents were subjected to 3 pump/purge cycles with N_2 followed by addition of anhydrous, degassed toluene or chlorobenzene (5 mL) via syringe. The reaction mixture was stirred at 110 °C for three days. After cooling to room temperature, the deeply purple or green colored reaction mixture was dropped into 100 mL vigorously stirred methanol (containing 5 mL 1 M hydrochloride acid). After stirring for 4 hours, the precipitated solid was collected by filtration. The solid polymers were redissolved in chloroform and reprecipitated into methanol. After filtration, the polymers were subjected to sequential Soxhlet extraction. The sequential solvents

were methanol, hexane and chloroform. Impurities and low-molecular-weight fraction were removed by methanol and hexane. Finally, the polymer solution in chloroform was concentrated to give the target polymer as a purple or green dark solid.

PNDITEG-AH[S4] Synthesis according to the general polymerization procedure: monomer NDITEG (74 mg, 0.1 mmol), monomer AH (77 mg, 0.1 mmol), dry chlorobenzene (5 mL). The polymer was obtained as a purple solid (76 mg, 74 %). ^{1}HNMR (400 MHz, $CDCl_3$): δ: 8.91-8.71 (m, 2H), 7.31-7.26 (m, 2H), 4.52-4.36 (m, 4H), 3.92-3.39 (m, 28H), 2.79-2.53 (m, 4H) 1.78-1.62 (m, 4H), 1.47-1.19 (m, 32H), 1.19-1.09 (m, 8H), 0.95-0.80 (m, 6H). IR (cm^{-1}): 661, 724, 764, 793, 838, 929, 1110, 1208, 1255, 1308, 1348, 1378, 1436, 1575, 1666, 1704, 2852, 2921.

PNDITEG-TVT Synthesis according to the general polymerization procedure: monomer NDITEG (74 mg, 0.1 mmol), monomer TVT (80 mg, 0.1 mmol), dry chlorobenzene (5 mL). The polymer was obtained as a green solid (92 mg, 87 %). ^{1}HNMR (400 MHz, $CDCl_3$): δ: 8.81-8.76 (m, 2H), 7.20-7.07 (m, 4H), 4.51-4.37 (m, 4H), 3.92-3.43 (m, 28H), 2.81-2.71 (m, 4H) 1.78-1.65 (m, 8H), 1.53-1.04 (m, 40H), 0.93-0.80 (m, 6H). IR (cm^{-1}): 656, 720, 764, 791, 864, 919, 1106, 1180, 1204, 1246, 1274, 1311, 1435, 1567, 1665, 1703, 2851, 2920.

PNDIC8TEG-TVT Synthesis according to the general polymerization procedure: monomer NDIC8TEG (74 mg, 0.1 mmol), monomer TVT (80 mg, 0.1 mmol), dry chlorobenzene (5 mL). The polymer was obtained as a green solid (115 mg, 90 %). ^{1}HNMR (400 MHz, $CDCl_3$): δ: 8.84-8.73 (m, 2H), 7.20-7.06 (m, 4H), 4.20-4.04 (m, 4H), 3.72-3.32 (m, 44H), 2.82-2.64 (m, 4H) 1.81-1.19 (m, 80H), 0.92-0.81 (m, 6H). IR (cm^{-1}): 721, 765, 793, 853, 919, 1105, 1180, 1241, 1273, 1312, 1367, 1455, 1567, 1662, 1701, 2851, 2920.

3 ^{1}HNMR of Polymers

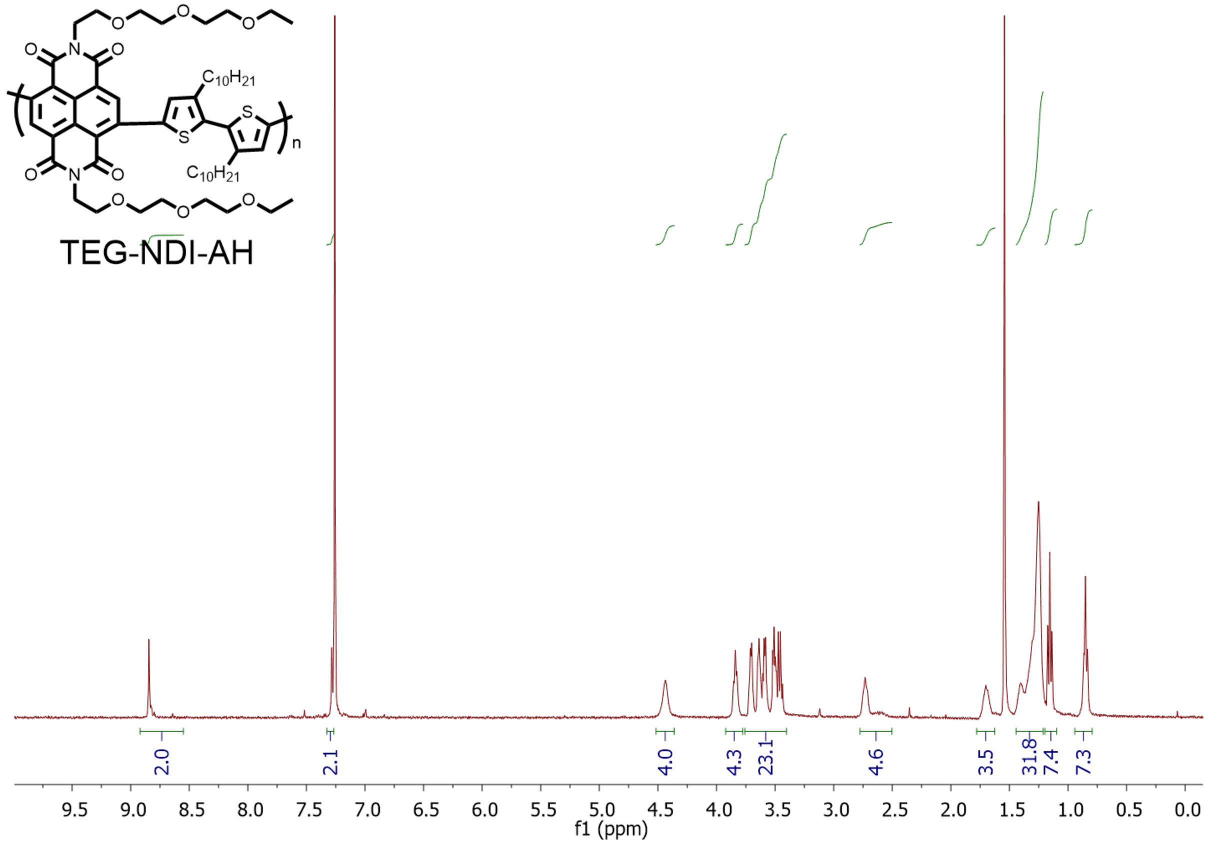

Figure S16: The ^{1}HNMR spectra of **PNDITEG-AH** (Adapted from our previous work[S4])

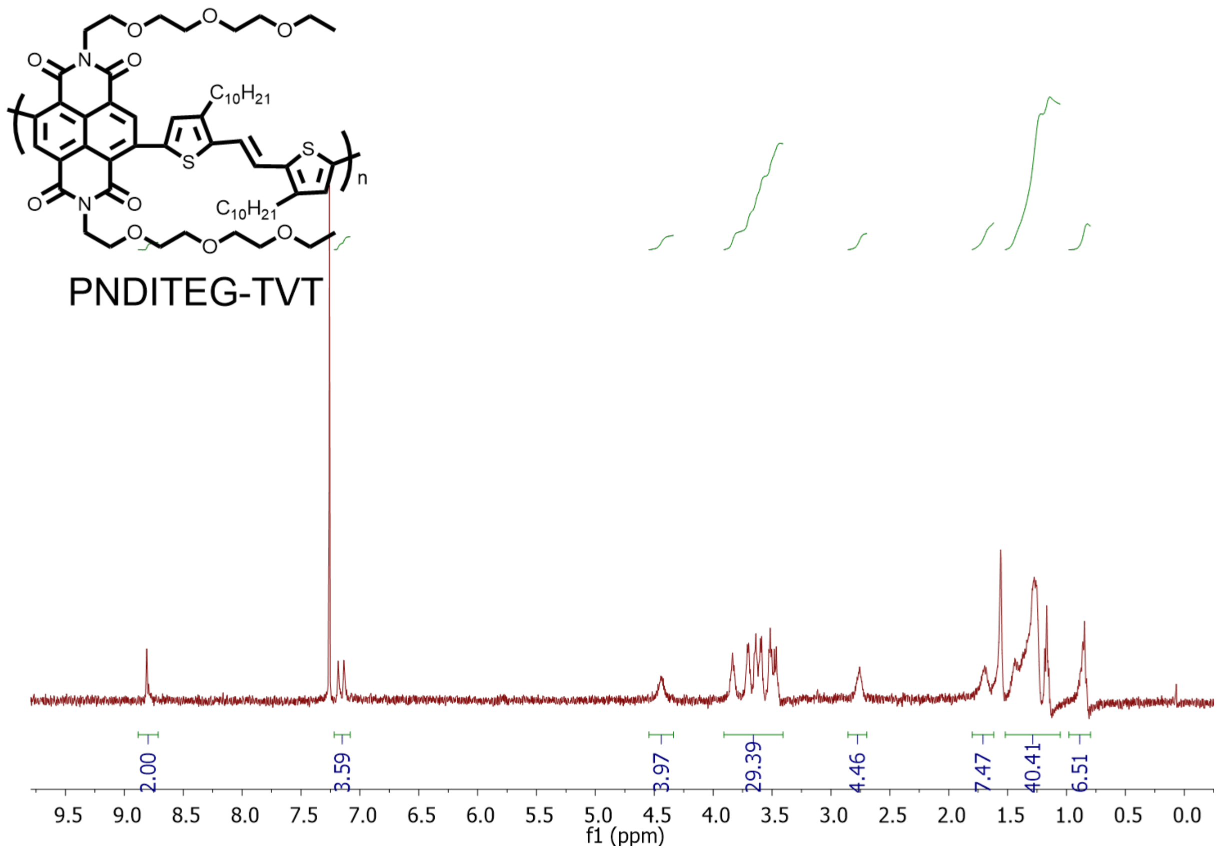

Figure S17: The ^{1}HNMR spectra of **PNDITEG-TVT**

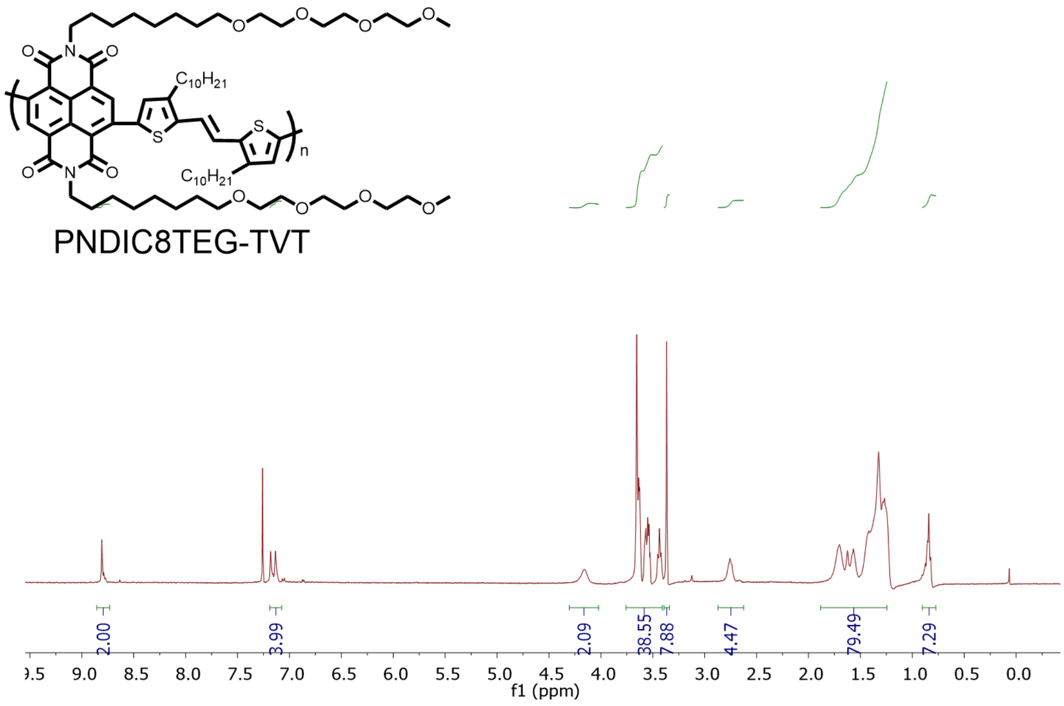

Figure S18: The ^{1}HNMR spectra of **PNDIC8TEG-TVT**

4 Fourier transform infrared spectroscopy (FT-IR)

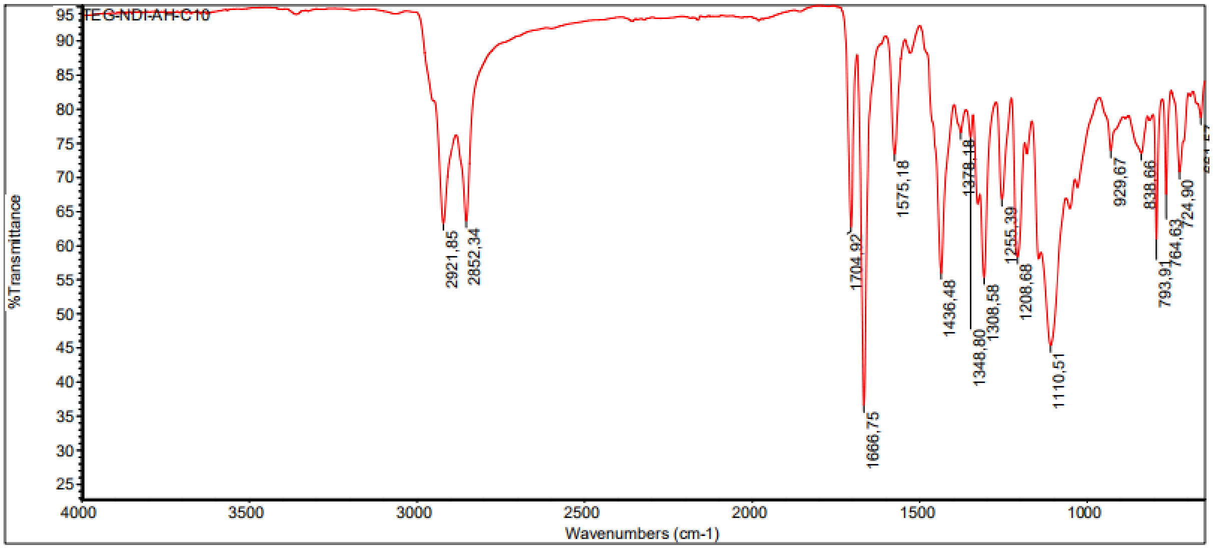

Figure S19: The IR spectra of **PNDITEG-AH** (Adapted from our previous work[S4])

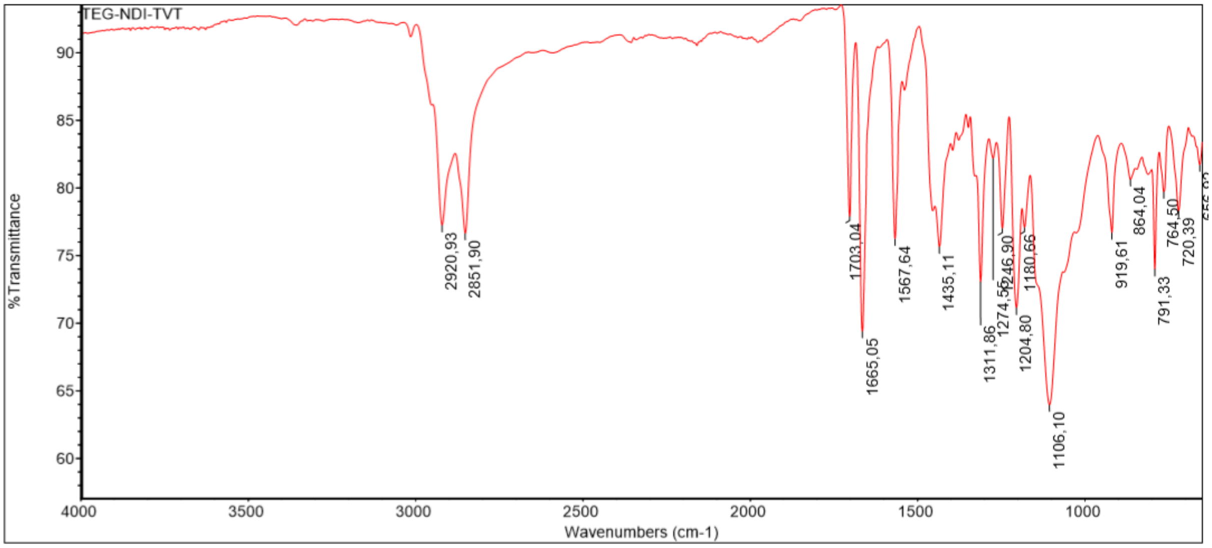

Figure S20: The IR spectra of **PNDITEG-TVT**

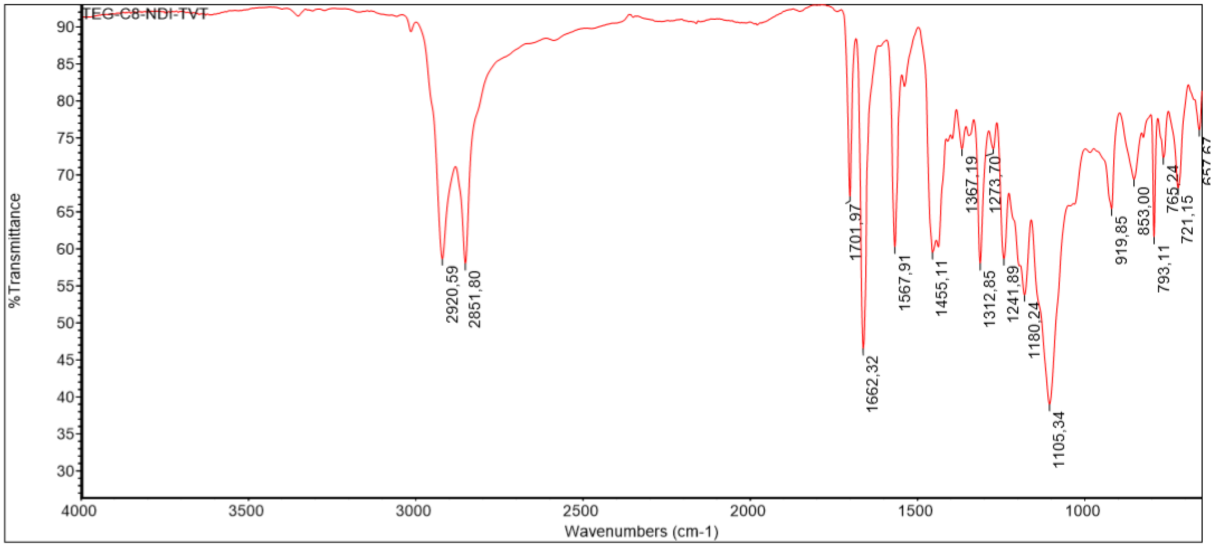

Figure S21: The IR spectra of **PNDIC8TEG-TVT**

5 Gel Permeation Chromatography (GPC)

Table S1: The molecular weight of **PNDITEG-AH**, **PNDITEG-TVT** and **PNDIC8TEG-TVT**.

Polymer	M_n (g mol^{-1})	M_W (g mol^{-1})	PDI
**PNDITEG-AH**	9014	14568	1.62
**PNDITEG-TVT**	14246	54572	3.83
**PNDIC8TEG-TVT**	15662	55990	3.57

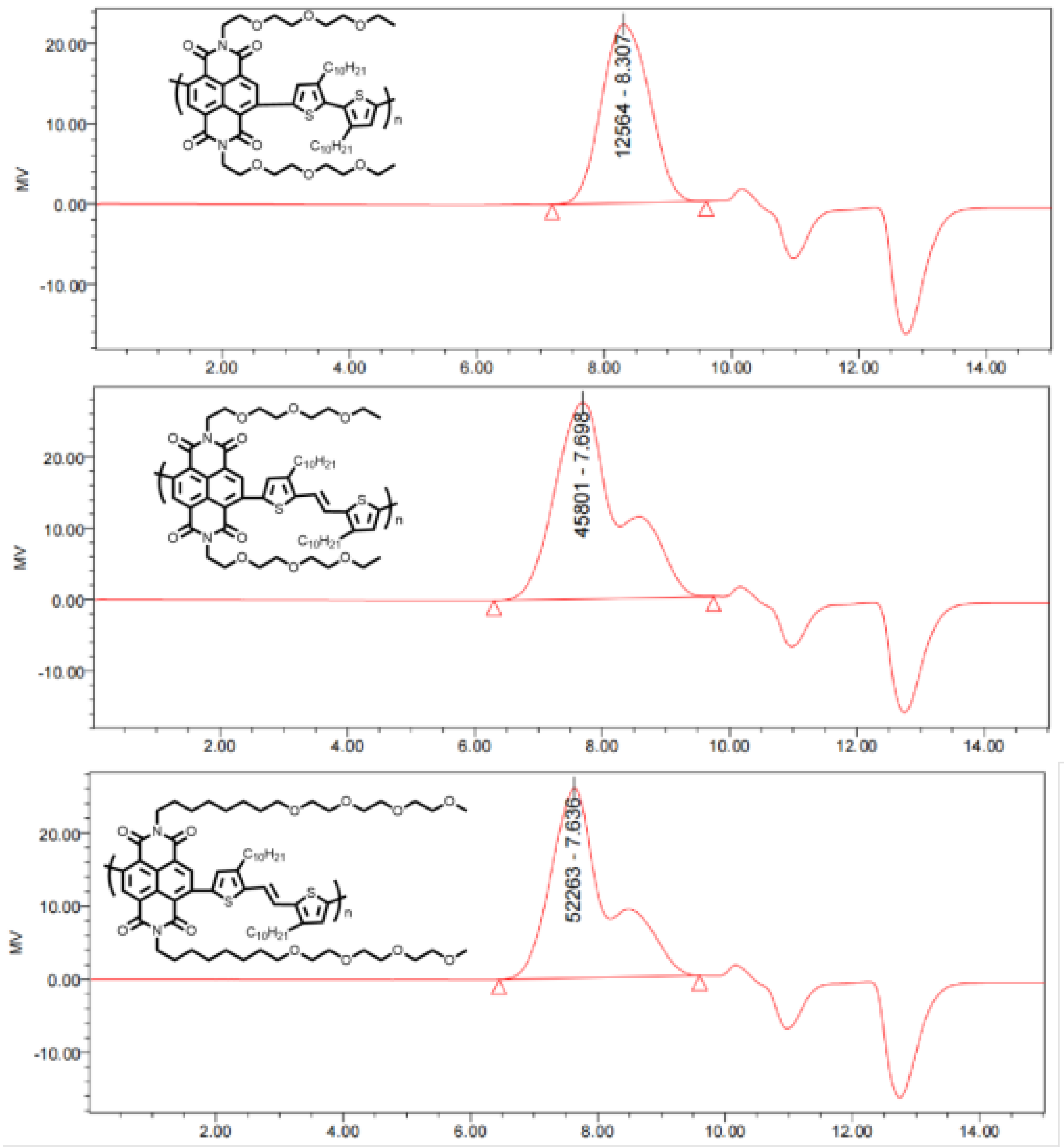

Figure S22: The GPC data of **PNDITEG-AH**, **PNDITEG-TVT** and **PNDIC8TEG-TVT**

6 Matrix-assisted laser desorption/ionization time-of-flight mass (MALDI-TOF-MS)

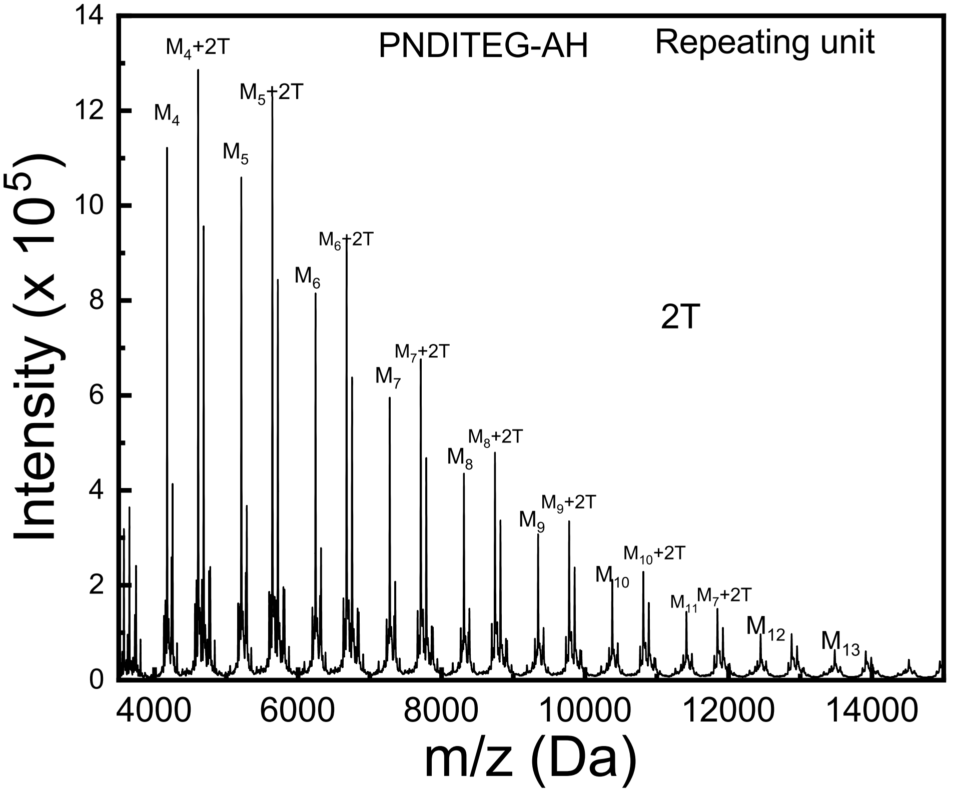

Figure S23: The MALDI-TOF-MS spectra of **PNDITEG-AH**

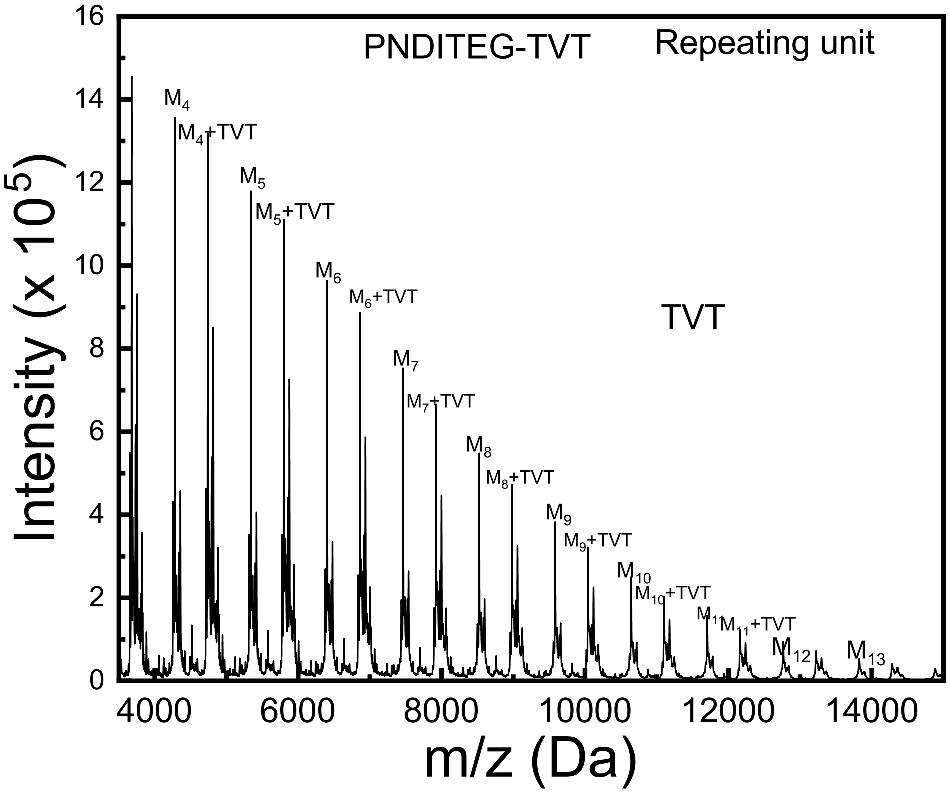

Figure S24: The MALDI-TOF-MS spectra of **PNDITEG-TVT**

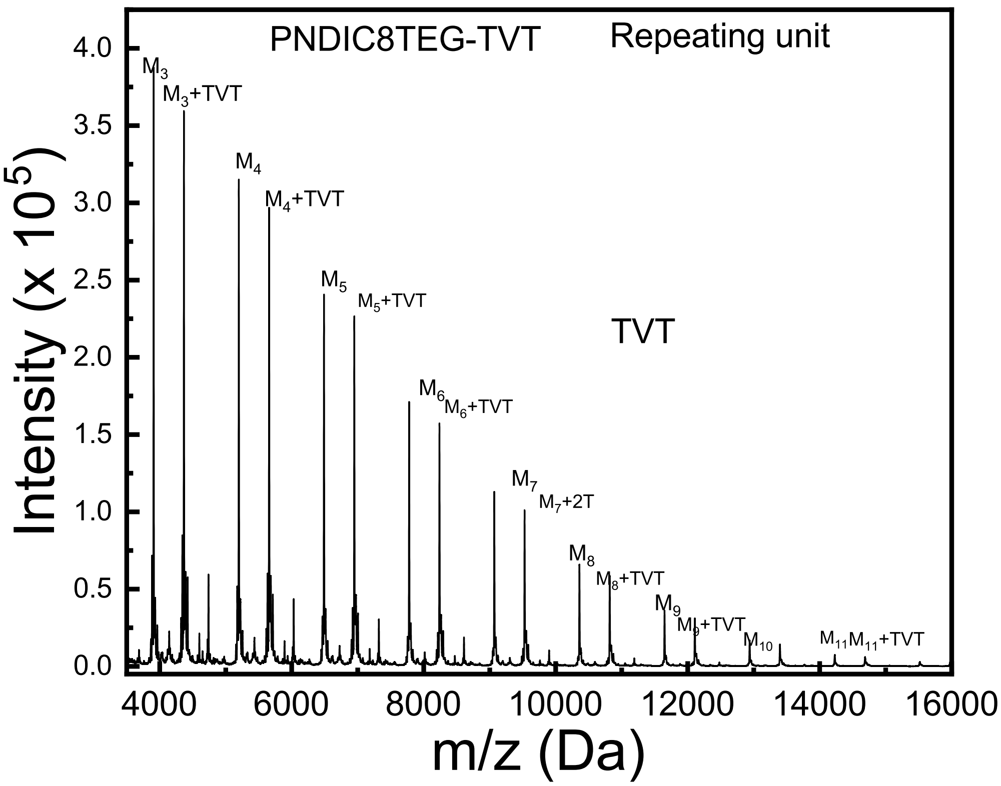

Figure S25: The MALDI-TOF-MS spectra of **PNDIC8TEG-TVT**

7 Thermal Properties

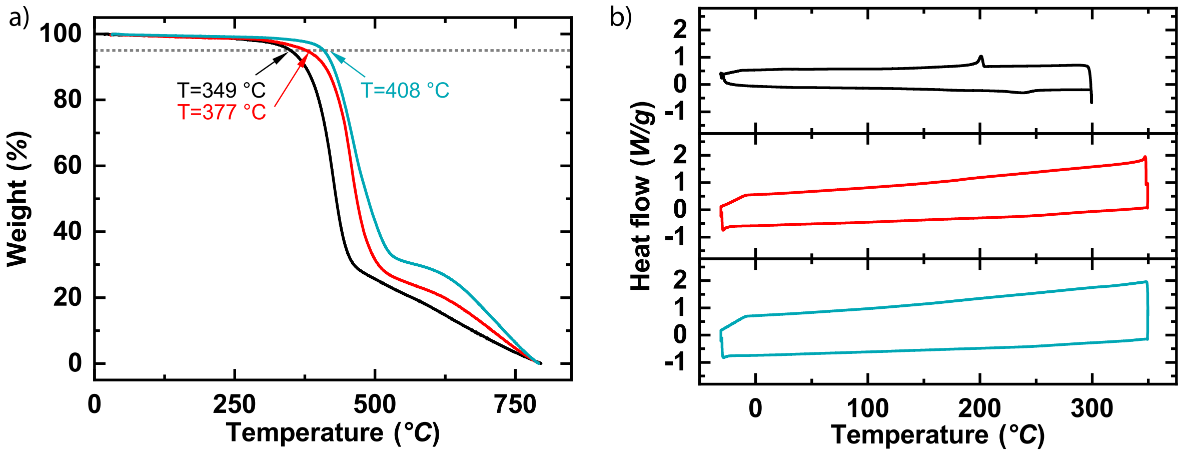

Figure S26: Thermal properties for the polymers **PNDITEG-AH** (black), **PNDITEG-TVT** (red) and **PNDIC8TEG-TVT** (blue). (a) Thermogravimetric analysis measured at a rate of 20°C/min. (b) DSC analysis on the second heating cycle (first time cooling) measured at a rate of

10°C/min.

8 Optical Properties

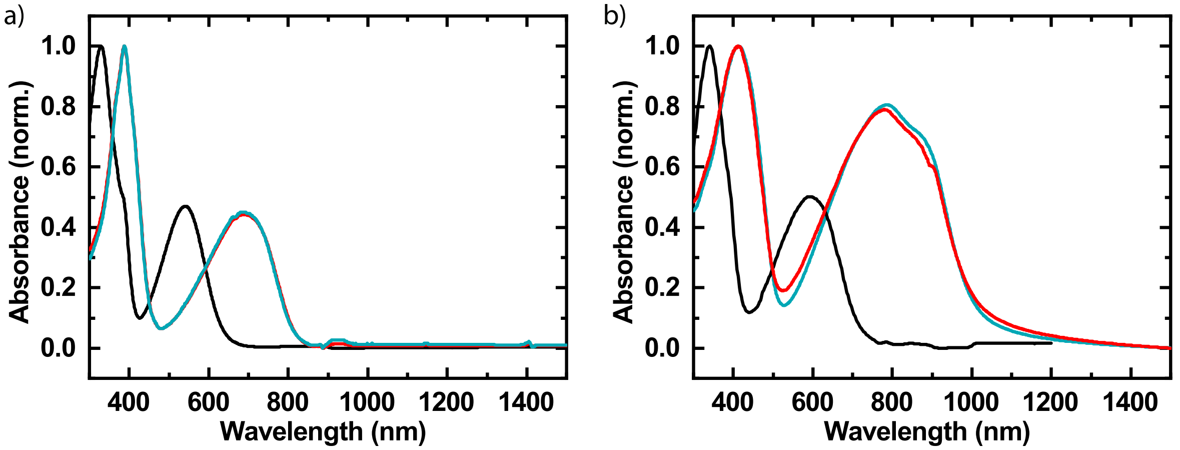

Figure S27: Normalized absorption spectra of pristine **PNDITEG-AH** (black), **PNDITEG-TVT** (red) and **PNDIC8TEG-TVT** (blue) in (a) dilute (10^{-5} M) solution phase as dissolved in $CHCl_3$ and (b) as drop-cast films.

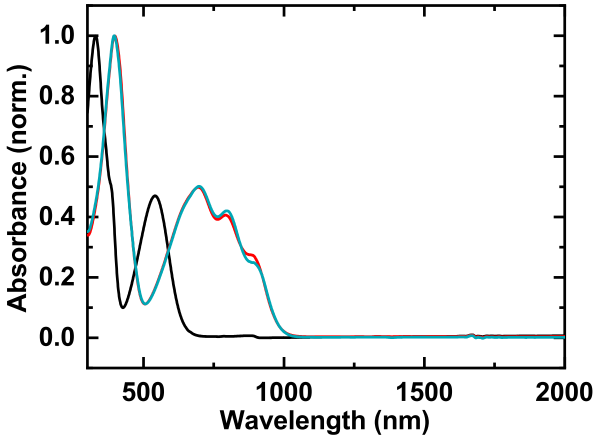

Figure S28: Absorption spectra of 3 mg of **PNDITEG-AH** (black), **PNDITEG-TVT** (red) and **PNDIC8TEG-TVT** (blue) in 10 ml toluene as used for wrapping HiPCO SWCNT

9 Density Functional Theory Calculation

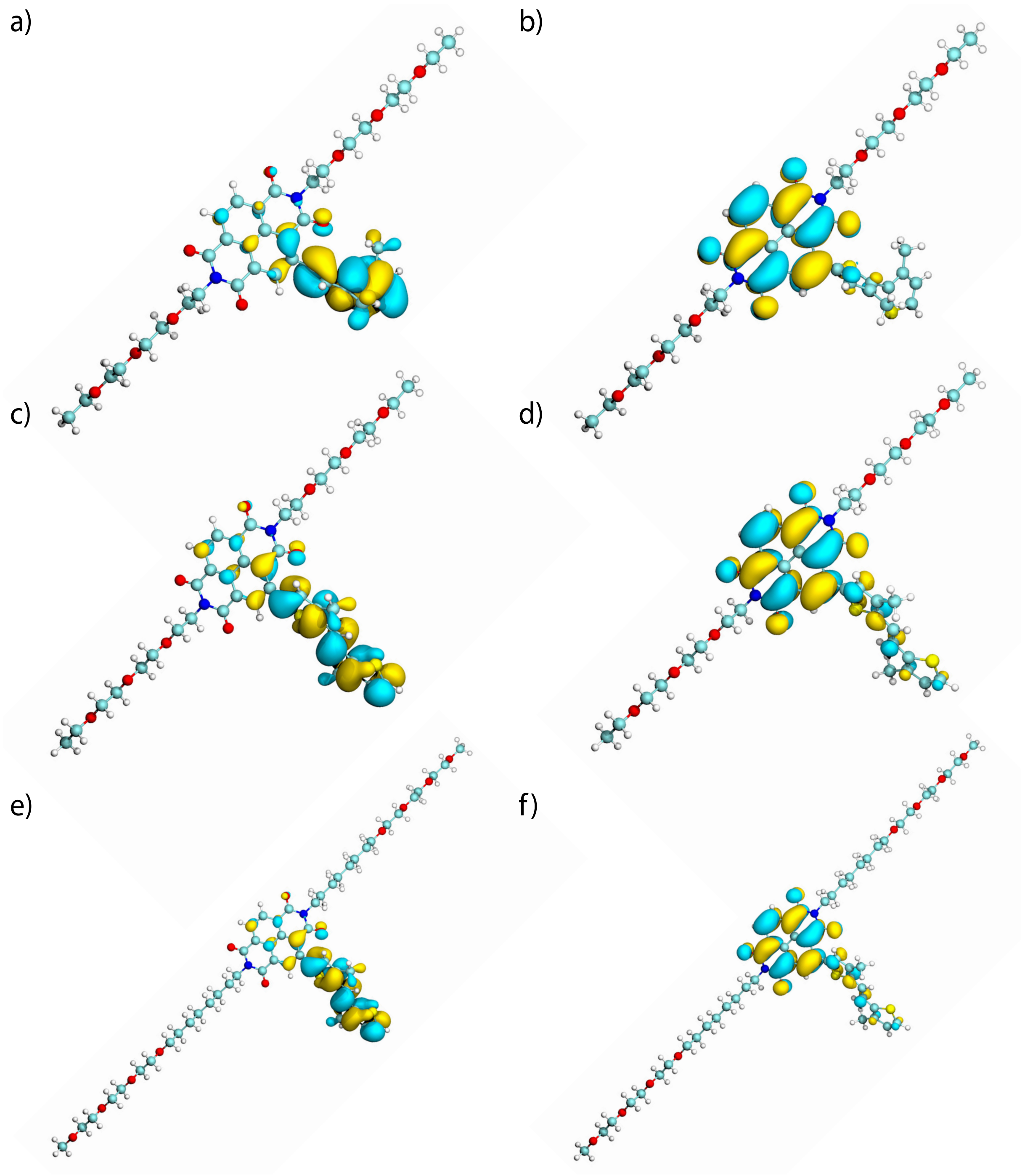

Figure S29: DFT calculated HOMO (left-side panels) and LUMO (right-side panels) for **PNDITEG-AH** (a, b) (Data from our previous work[S4]), **PNDITEG-TVT** (c, d), and **PNDIC8TEG-TVT** (e, f).

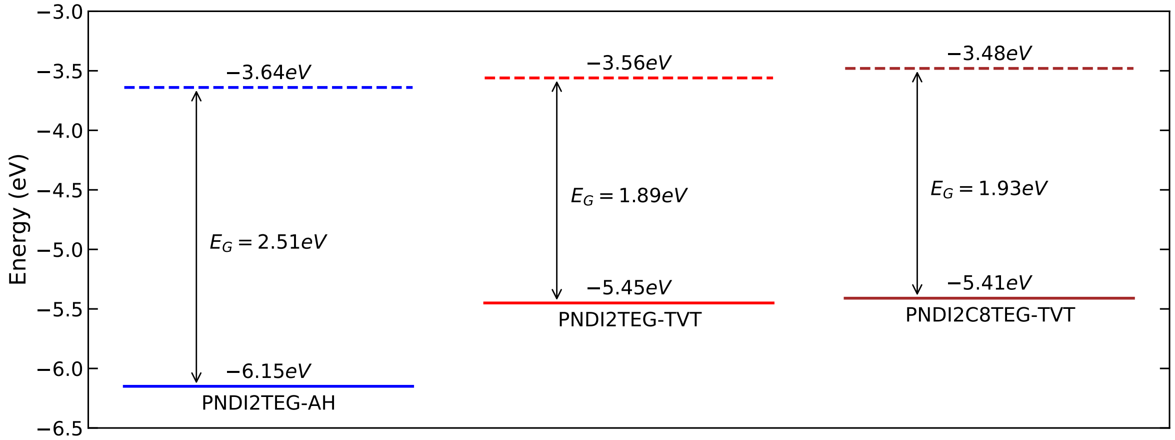

Figure S30: HOMO and LUMO energy levels and their respective bandgaps for **PNDITEG-AH**, **PNDITEG-TVT**, and **PNDIC8TEG-TVT**.

Table S2: Dihedral angles in **PNDITEG-AH**, **PNDITEG-TVT**, and **PNDIC8TEG-TVT**

Polymer	θ_1	θ_2	θ_3
**PNDITEG-AH**	61.9°	109°	
**PNDITEG-TVT**	39.5°	4.8°	1.7°
**PNDIC8TEG-TVT**	40.1°	4.7°	1.6°

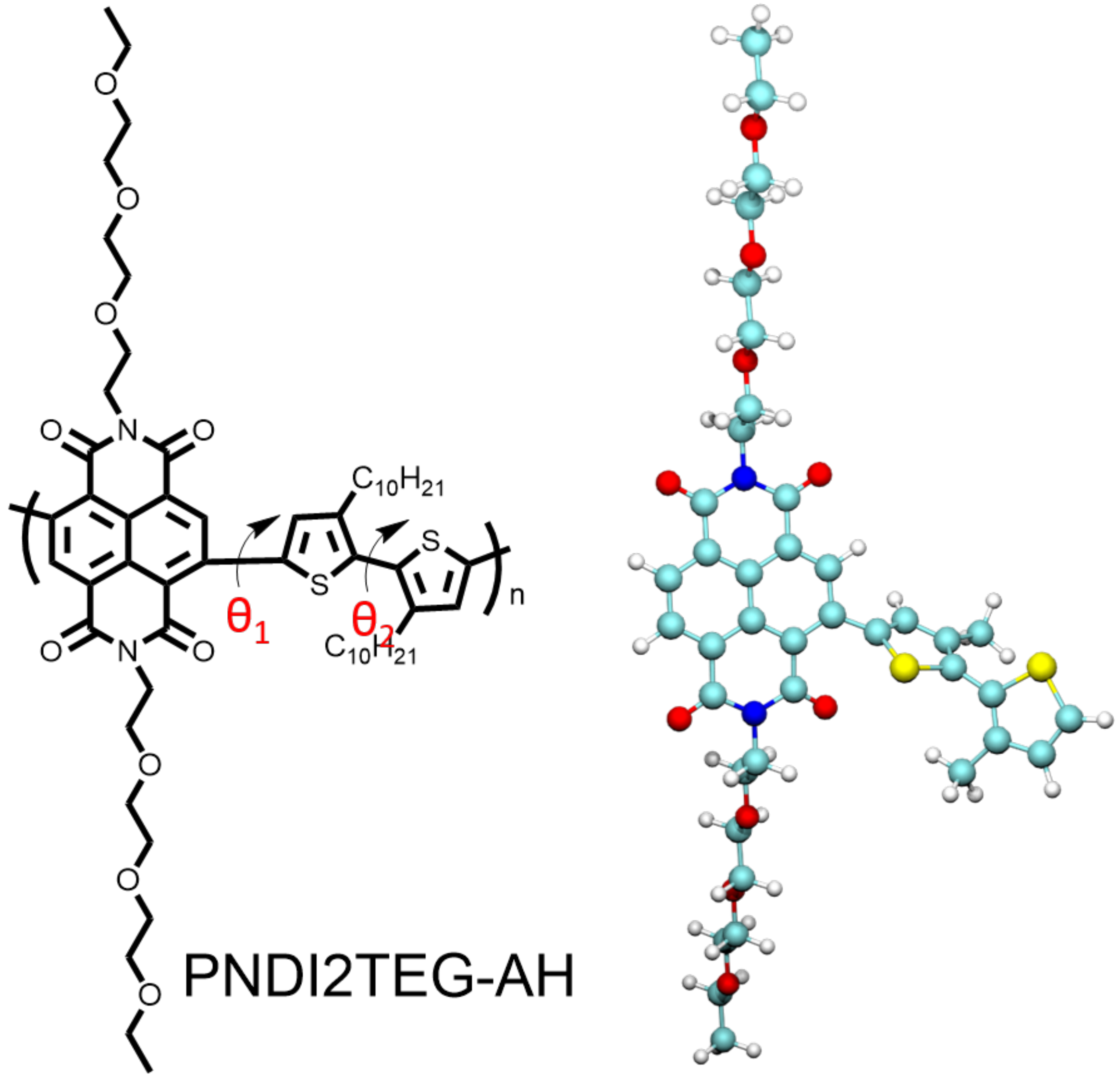

Figure S31: Optimized structure of **PNDITEG-AH**(Data from our previous work[S4])

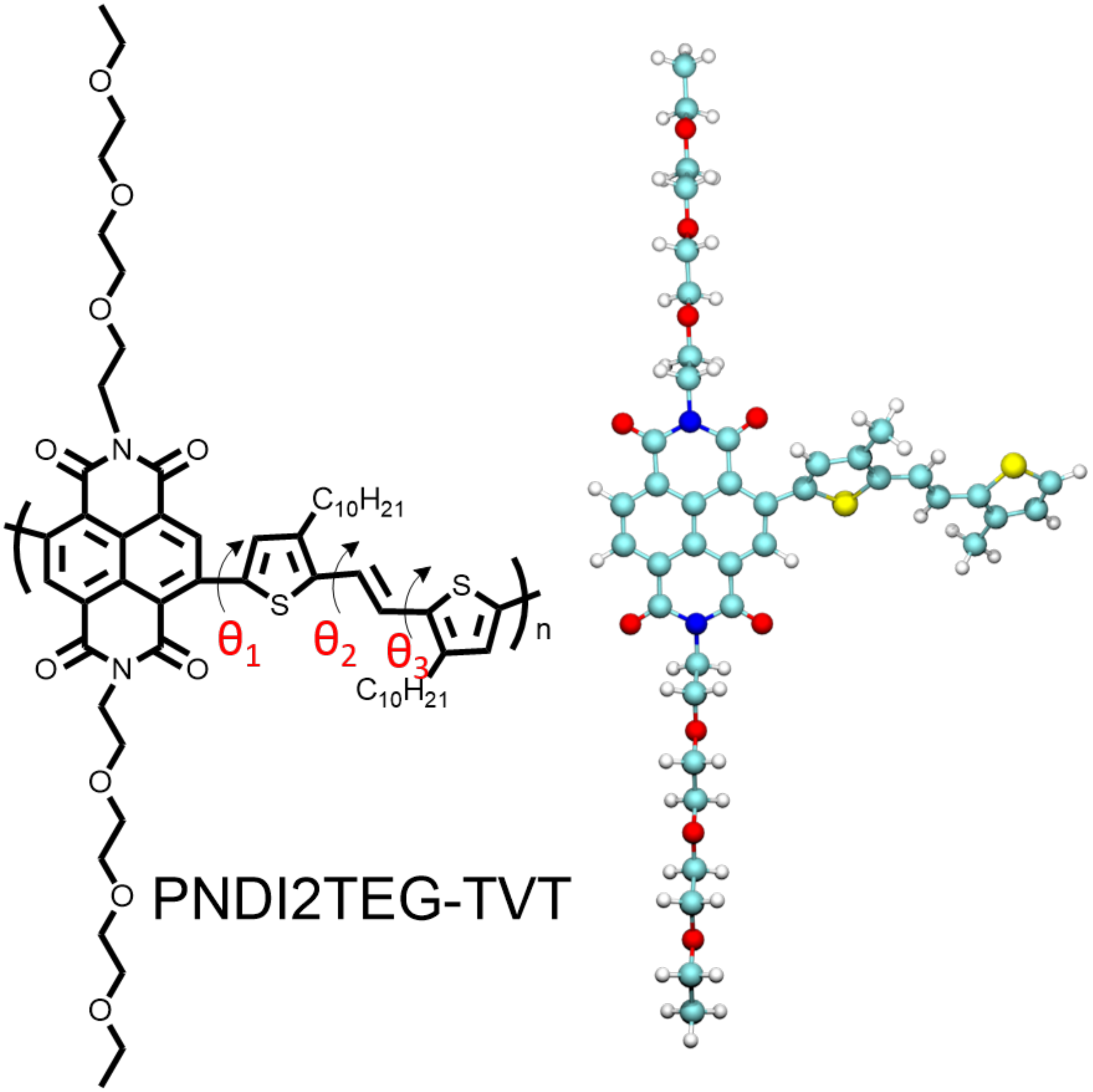

Figure S32: Optimized structure of **PNDITEG-TVT**

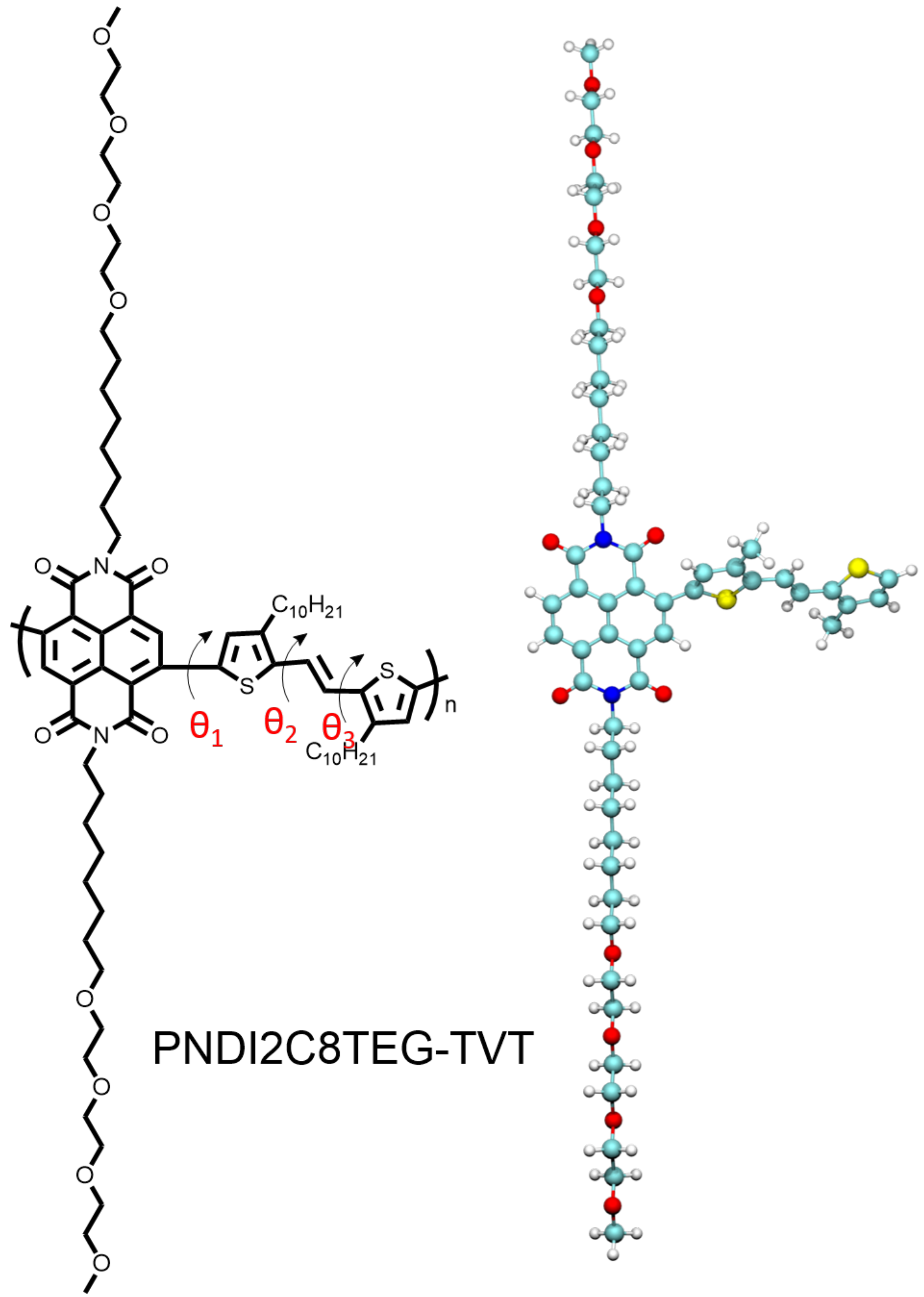

Figure S33: Optimized structure of **PNDIC8TEG-TVT**

10 Preparation and characterization of s-SWCNT dispersions

HiPCO SWCNT (0.8 nm to 1.2 nm) were purchased from Unidym Inc. First the polymer was solubilized in toluene, subsequently SWCNT was added to form the SWCNT:polymer dispersion at a weight ratio of 1 : 2 (3 mg of SWCNT, 6 mg of polymer, 15 ml of toluene), the solution was then sonicated in a cup-horn for 2 h at 78 W. After ultrasonication, the dispersion was centrifuged at 30 000 rpm (109 000 g) for 1 h in an ultracentrifuge (Beckman Coulter Optima XE-90; rotor: SW55Ti). After the centrifugation, the highest density components precipitated at the bottom of the centrifugation tube, while the low-density components, including small bundles and individualized SWCNTs wrapped by the polymer, and free polymer chains, remained in the upper part as supernatant. One extra step of ultracentrifugation was implemented to decrease the amount of excess polymer in solution (enrichment). In this step the supernatant obtained after the first ultracentrifugation was centrifuged for 5 h at 55 000 rpm (367 000 g). At this point, the individualized s-SWCNTs precipitated to form a pellet, and the free polymer remained in the supernatant. Finally, the pellet was taken away and re-dispersed by sonication in o-xylene as described in previous work.[S14]

11 Optical Characterization of the s-SWCNT Dispersion

Optical measurements were performed to check the concentration of semiconducting carbon nanotubes selected by the polymer and to monitor the colloidal stability of the ink over time. Absorption spectra were recorded with a UV–vis–NIR spectrophotometer (Shimadzu UV-3600). For the PL measurements the samples were excited at ≈800 nm by the fundamental mode of a mode-locked Ti:Sapphire laser (Mira 900, Coherent). A variable neutral density filter was used to adjust the laser excitation power on the sample. The PL decays were

recorded with an NIR sensitive Hamamatsu streak camera working in synchroscan mode (time resolution ≈2 ps). The steady-state PL spectra were measured with an InGaAs Andor inline detector, and corrected for the spectral response of the setup using a calibrated light source. Fitting and Data Analysis for Optical Measurements: Steady-state spectra were fitted with Gaussian functions (the average goodness-of-fit was determined by the chi-squared (χ^2) test). Lifetimes were fitted using exponential functions such as $I = \sum iA_i \exp \frac{-t}{\tau_i}$. When only one lifetime was reported, the best fit could be achieved with a monoexponential function; otherwise a bi-exponential function was used.

The results of Raman spectroscopy are displayed in **figure S34**. The D-mode was found to be at 1384 cm^{-1}, G^+-mode at 1582 cm^{-1}, and G^--mode at 1606 cm^{-1}. Also, the RBM for semiconductive species were found in the region of 150 cm^{-1} to 210 cm^{-1}. Evidence was found for presence of bundles or metallic species at 210 cm^{-1} to 280 cm^{-1}, with similar amount for **PNDITEG-TVT** and **PNDIC8TEG-TVT** but slightly more for **PNDITEG-AH**. Since over time bundles are formed in the aged ink, a low $\frac{G}{D}$-ratio is visible as indicator for the ink quality. However, no evidence is seen for a significant presence of metallic species. The $\frac{G^+}{G^-}$-ratio is indicative of the chiral angle of the SWCNT.[S15] This ratio is similar, but slightly increasing (1.16, 1.50 and 1.67) going from **PNDITEG-AH** to **PNDITEG-TVT** and **PNDIC8TEG-TVT**. Therefore, we can conclude that all polymers pick up mostly similar species, but that **PNDITEG-AH** has a slight preference for picking up species of lower, and **PNDIC8TEG-TVT** of higher chiral angle as compared with **PNDITEG-TVT**.

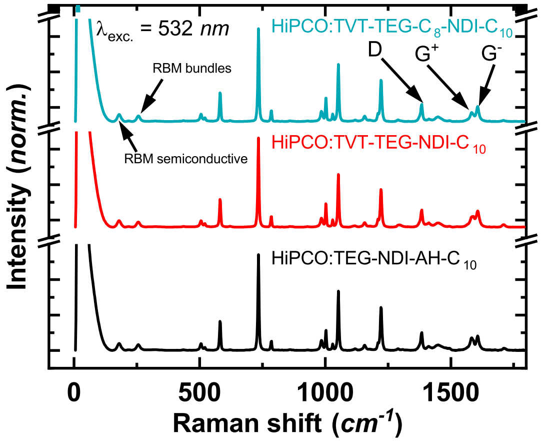

Figure S34: Raman spectroscopy of the inks HiPCO:**PNDITEG-AH** (black), HiPCO:**PNDITEG-TVT** (red) and HiPCO:**PNDIC8TEG-TVT** (turquoise)

.

12 FET transistor fabrication and electrical characterization

Field-effect transistors were fabricated on silicon substrates with on top a thermally grown SiO_2 dielectric layer (230 nm thickness, and a dielectric constant of 3.9). Source and drain bottom electrodes (10 nm ITO/30 nm Au) were lithographically patterned, forming an interdigitated channel. The different s-SWCNT dispersions tested were deposited in a nitrogen-filled glovebox by blade coating (Zehntner ZAA 2300 Automatic film applicator coater). The deposition procedure was repeated two times to achieve a sufficiently high s-SWCNT coverage density. After deposition, the samples were annealed at 160 °C for 60 min to evaporate the remaining solvent.

Electrical measurements were performed using a probe station placed in a nitrogen-filled glovebox at room temperature under dark conditions, unless otherwise specified. The probe station is connected to an Agilent E5270B Semiconductor Parameter Analyzer. The reported charge carrier mobilities were extracted from the $I_{DS} - V_G$ transfer characteristics in the linear regime at $V_D = \pm 5$ V. Unless otherwise mentioned, the channel length (L) and

width (W) are 20 µm and 2 mm, respectively. The gate capacitance is 15 nF cm^{-2}.

Table S3: Summary of the FET data averaged over all samples for inks obtained after selection (1st) and after enrichment (2nd).

	**PNDITEG-AH** 1st	**PNDITEG-AH** 2nd	**PNDITEG-TVT** 1st	**PNDITEG-TVT** 2nd	**PNDIC8TEG-TVT** 1st	**PNDIC8TEG-TVT** 2nd
**on/off** ($\boldsymbol{h^+}$)	2×10^7	4×10^6	2×10^5	8×10^6	2×10^4	7×10^5
**on/off** ($\boldsymbol{e^-}$)	1×10^3	4×10^3	1×10^4	3×10^5	1×10^4	7×10^5
μ ($\boldsymbol{h^+}$) (**cm^2 V^{-1} s^{-1}**)	2×10^{-2}	0.6	2×10^{-3}	1.0	2×10^{-5}	1.6
μ ($\boldsymbol{e^-}$) (**cm^2 V^{-1} s^{-1}**)	1×10^{-5}	1×10^{-2}	8×10^{-5}	2×10^{-2}	1×10^{-4}	0.2
V_{th} ($\boldsymbol{h^+}$) ($\boldsymbol{V}$)	-2.0	-29.7	-40.0	-44.9	-27.1	-43.8
V_{th} ($\boldsymbol{e^-}$) ($\boldsymbol{V}$)	35.4	15.0	15.5	1.1	-0.6	-8.9
Hys ($\boldsymbol{e^-}$) ($\boldsymbol{V}$)	-	-10.7	-	-12.4	-	-11.4
Hys ($\boldsymbol{h^+}$) ($\boldsymbol{V}$)	-	13.4	-	3.4	-	3.3

13 Atomic Force Microscopy (AFM) image of FET device

Device morphologies are probed by means of atomic force microscopy (AFM). Typical morphologies are depicted in **figure S35**, which demonstrate that SWCNTs networks form well-percolated semiconducting layers.

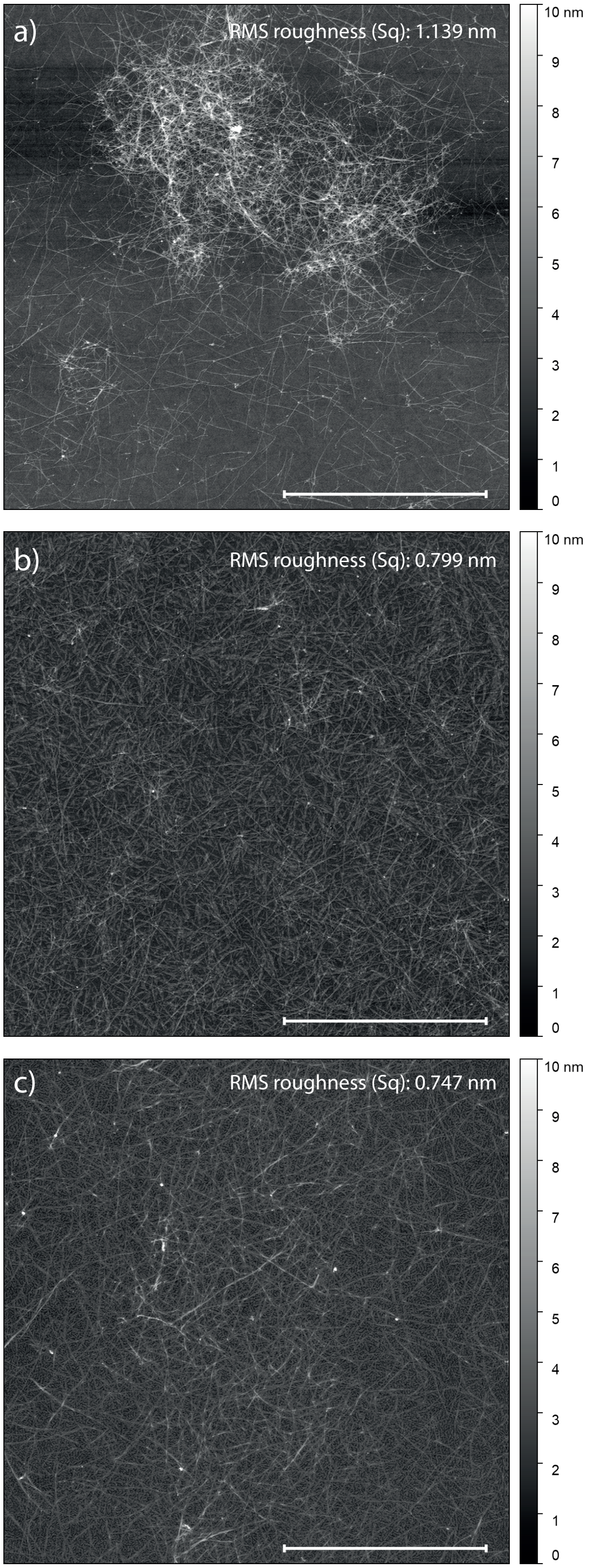

Figure S35: AFM images of the FET channel area showing the random s-SWCNT network for HiPCO:**PNDITEG-AH** (a), (a) HiPCO:**PNDITEG-TVT** and (b) HiPCO:**PNDIC8TEG-TVT**. The inset scale bar is 2 µm.

References

(S1) Brédas, J. L.; Silbey, R.; Boudreaux, D. S.; Chance, R. R. Chain-Length Dependence of Electronic and Electrochemical Properties of Conjugated Systems: Polyacetylene, Polyphenylene, Polythiophene, and Polypyrrole. *Journal of the American Chemical Society* **1983**, *105*, 6555–6559.

(S2) Kucur, E.; Riegler, J.; Urban, G. A.; Nann, T. Determination of quantum confinement in CdSe nanocrystals by cyclic voltammetry. *Journal of Chemical Physics* **2003**, *119*, 2333–2337.

(S3) Frisch, M. J.; Trucks, G. W.; Schlegel, H. B.; Scuseria, G. E.; Robb, M. A.; Cheeseman, J. R.; Scalmani, G.; Barone, V.; Petersson, G. A.; Nakatsuji, H.; Li, X.; Caricato, M.; Marenich, A. V.; Bloino, J.; Janesko, B. G.; Gomperts, R.; Mennucci, B.; Hratchian, H. P.; Ortiz, J. V.; Izmaylov, A. F.; Sonnenberg, J. L.; Williams-Young, D.; Ding, F.; Lipparini, F.; Egidi, F.; Goings, J.; Peng, B.; Petrone, A.; Henderson, T.; Ranasinghe, D.; Zakrzewski, V. G.; Gao, J.; Rega, N.; Zheng, G.; Liang, W.; Hada, M.; Ehara, M.; Toyota, K.; Fukuda, R.; Hasegawa, J.; Ishida, M.; Nakajima, T.; Honda, Y.; Kitao, O.; Nakai, H.; Vreven, T.; Throssell, K.; Montgomery, J. A., Jr.; Peralta, J. E.; Ogliaro, F.; Bearpark, M. J.; Heyd, J. J.; Brothers, E. N.; Kudin, K. N.; Staroverov, V. N.; Keith, T. A.; Kobayashi, R.; Normand, J.; Raghavachari, K.; Rendell, A. P.; Burant, J. C.; Iyengar, S. S.; Tomasi, J.; Cossi, M.; Millam, J. M.; Klene, M.; Adamo, C.; Cammi, R.; Ochterski, J. W.; Martin, R. L.; Morokuma, K.; Farkas, O.; Foresman, J. B.; Fox, D. J. Gaussian˜16 Revision C.01. 2016; Gaussian Inc. Wallingford CT.

(S4) Ye, G.; Talsma, W.; Tran, K.; Liu, Y.; Dijkstra, S.; Cao, J.; Chen, J.; Qu, J.; Song, J.; Loi, M. A.; Chiechi, R. C. Polar Side Chains Enhance Selection of Semiconducting Single-Walled Carbon Nanotubes by Polymer Wrapping. *Macromolecules* **2022**, *55*, 1386–1397.

(S5) Guo, X.; Watson, M. D. Conjugated Polymers from Napthalene Bisimide. *Organic Letters* **2008**, *10*, 5333–5336.

(S6) Higginbotham, H. F.; Maniam, S.; Langford, S. J.; Bell, T. D. New brightly coloured, water soluble, core-substituted naphthalene diimides for biophysical applications. *Dyes and Pigments* **2015**, *112*, 290–297.

(S7) Komáromy, D.; Stuart, M. C.; Monreal Santiago, G.; Tezcan, M.; Krasnikov, V. V.; Otto, S. Self-assembly can direct dynamic covalent bond formation toward diversity or specificity. *Journal of the American Chemical Society* **2017**, *139*, 6234–6241.

(S8) Kim, R.; Kang, B.; Sin, D. H.; Choi, H. H.; Kwon, S.-K.; Kim, Y.-H.; Cho, K. Oligo (ethylene glycol)-incorporated hybrid linear alkyl side chains for n-channel polymer semiconductors and their effect on the thin-film crystalline structure. *Chemical Communications* **2015**, *51*, 1524–1527.

(S9) Liu, J.; Ye, G.; Potgieser, H. G. O.; Koopmans, M.; Sami, S.; Nugraha, M. I.; Villalva, D. R.; Sun, H.; Dong, J.; Yang, X.; Qiu, X.; Yao, C.; Portale, G.; Fabiano, S.; Anthopoulos, T. D.; Baran, D.; Havenith, R. W. A.; Chiechi, R. C.; Koster, L. J. A. Amphipathic Side Chain of a Conjugated Polymer Optimizes Dopant Location toward Efficient N-Type Organic Thermoelectrics. *Advanced Materials* **2021**, *33*, 2006694.

(S10) He, M.; Leslie, T. M.; Sinicropi, J. A. Synthesis of Chromophores with Extremely High Electro-optic Activity. 1. Thiophene-Bridge-Based Chromophores. *Chemistry of Materials* **2002**, *14*, 4662–4668.

(S11) Yamamoto, T.; Kumagai, A.; Kokubo, H.; Nakamura, Y. π-Conjugated Polymers, (Th(R)–CH=CH–Th(R))n (Th(R) = 3-Alkylthiophene-2,5-diyl; R = Octyl, Decyl, and Dodecyl): Preparation of Dibromo Monomers and the Polymers. *Bulletin of the Chemical Society of Japan* **2011**, *84*, 1291–1293.

(S12) Lee, M.-H.; Kim, J.; Kang, M.; Kim, J.; Kang, B.; Hwang, H.; Cho, K.; Kim, D.-Y. Precise Side-Chain Engineering of Thienylenevinylene–Benzotriazole-Based Conjugated Polymers with Coplanar Backbone for Organic Field Effect Transistors and CMOS-like Inverters. *ACS Applied Materials & Interfaces* **2017**, *9*, 2758–2766.

(S13) Heo, Y.-J.; Jeong, H.-G.; Kim, J.; Lim, B.; Kim, J.; Kim, Y.; Kang, B.; Yun, J.-M.; Cho, K.; Kim, D.-Y. Formation of Large Crystalline Domains in a Semiconducting Polymer with Semi-fluorinated Alkyl Side Chains and Application to High-Performance Thin-Film Transistors. *ACS Applied Materials & Interfaces* **2020**, *12*, 49886–49894.

(S14) Talsma, W.; Sengrian, A. A.; Salazar-Rios, J. M.; Duim, H.; Abdu-Aguye, M.; Jung, S.; Allard, S.; Scherf, U.; Loi, M. A. Remarkably Stable, High-Quality Semiconducting Single-Walled Carbon Nanotube Inks for Highly Reproducible Field-Effect Transistors. *Advanced Electronic Materials* **2019**, *5*, 1900288.

(S15) Saito, R.; Jorio, A.; Hafner, J. H.; Lieber, C. M.; Hunter, M.; McClure, T.; Dresselhaus, G.; Dresselhaus, M. S. Chirality-dependent G-band Raman intensity of carbon nanotubes. *Phys. Rev. B* **2001**, *64*, 085312.
